# Tropical cyclones and acute coronary syndromes: a Chinese nationwide study

**DOI:** 10.1093/eurheartj/ehag066

**Published:** 2026-02-18

**Authors:** Sicheng Li, Wei Wang, Hanwen Zhou, Yuqin Zhang, Lin Chen, Jiyi Lin, Nawsherwan, Yong Huo, Junbo Ge, Bin Wang, Yan Wang

**Affiliations:** Xiamen Cardiovascular Hospital of Xiamen University, School of Medicine, Fujian Branch of National Clinical Research Center for Cardiovascular Diseases, Xiamen University, Xiamen, Fujian, China; Department of Gastroenterology, Xinqiao Hospital, Third Military Medical University, Chongqing, China; School of Public Health, Chongqing Medical University, Chongqing, China; Peking University Center for Public Health and Epidemic Preparedness & Response, Peking University, Beijing, China; Department of Epidemiology and Health Statistics, West China School of Public Health/West China Fourth Hospital, Sichuan University, Chengdu, Sichuan, China; Xiamen Cardiovascular Hospital of Xiamen University, School of Medicine, Fujian Branch of National Clinical Research Center for Cardiovascular Diseases, Xiamen University, Xiamen, Fujian, China; Xiamen Chest Pain Quality Control Center, Xiamen, Fujian, China; Xiamen Cardiovascular Hospital of Xiamen University, School of Medicine, Fujian Branch of National Clinical Research Center for Cardiovascular Diseases, Xiamen University, Xiamen, Fujian, China; Department of Cardiology, Peking University First Hospital, Beijing, China; Department of Cardiology, Zhongshan Hospital, Fudan University, Shanghai Institute of Cardiovascular Diseases, Shanghai, China; Xiamen Cardiovascular Hospital of Xiamen University, School of Medicine, Fujian Branch of National Clinical Research Center for Cardiovascular Diseases, Xiamen University, Xiamen, Fujian, China; Xiamen Chest Pain Quality Control Center, Xiamen, Fujian, China; Xiamen Cardiovascular Hospital of Xiamen University, School of Medicine, Fujian Branch of National Clinical Research Center for Cardiovascular Diseases, Xiamen University, Xiamen, Fujian, China

**Keywords:** Extreme weather events, Cardiovascular diseases, Short-term risk, Developing countries, Environmental epidemiology, Climate change

## Abstract

**Background and Aims:**

Evidence on the associations between tropical cyclone (TC) exposure and acute coronary syndrome (ACS) remains limited, particularly in developing countries. Therefore, this study aimed to investigate the short-term association between TC exposure and ACS incidence and explore potential effect modifiers.

**Methods:**

This time-stratified case-crossover study included ACS patients from a nationwide registry in mainland China between 2015 and 2022. The Willoughby wind field model was chosen to estimate TC-associated wind speeds, with TC exposure defined as the occurrence of daily maximum sustained wind speeds ≥17.5 m/s. The outcomes included ACS and its subtypes, namely, ST-elevation myocardial infarction, non-ST-elevation myocardial infarction, and unstable angina. Conditional quasi-Poisson models with distributed lag non-linear models were applied to assess TC–ACS associations and lag structures. Subgroup analyses were conducted to identify potential effect modifiers.

**Results:**

A total of 2 563 780 individuals (64.0 ± 12.4 years; 68% males) were included. Compared with non-TC days, TC days were associated with longer delays in self-referral to the hospital (5.8 vs 5.3 h) and longer admission-to-catheterization times (1.0 vs 0.9 h). Over the 0–3-day period following TC exposure, the risk of developing ACS increased by 14% (95% confidence interval: 2% to 27%). Stronger associations were observed among males, individuals with lower education levels, and those with more ACS risk factors.

**Conclusions:**

TC exposure may increase the ACS burden by simultaneously increasing the risk of incidence and delaying treatment. The government, the public, and healthcare institutions must collaborate proactively to alleviate the burden of TC-associated ACS.

## Structured Graphical Abstract

**Structured Graphical Abstract:**
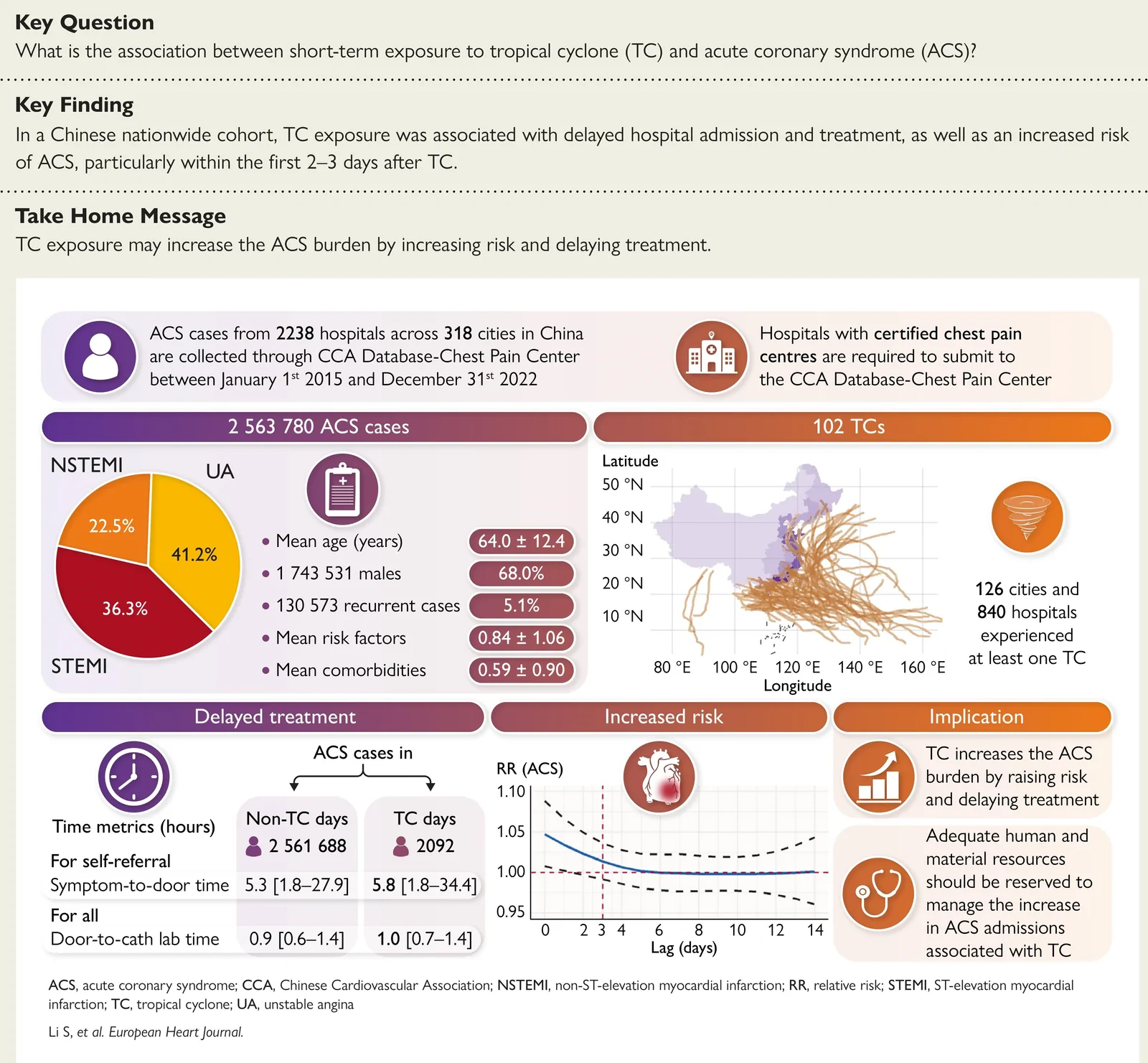

**See the editorial comment for this article ‘Preparing for the cardiovascular risks of tropical storms amplified by climate change’, by B.P. Geisler *et al*., https://doi.org/10.1093/eurheartj/ehag211.**

## Introduction

Tropical cyclones (TCs) are among the most destructive meteorological events and impose substantial social and economic burdens.^1^ For example, Hurricane Sandy alone resulted in economic losses of approximately USD 70 billion,^2^ and it is estimated that the average TC results in 7000–11 000 excess deaths in the United States.^3^ Nearly 560 million individuals are exposed to TCs each year worldwide.^4^ With ongoing global climate warming, the frequency^5^ and intensity^6^ of TCs, as well as the affected populations^4^ and the associated burden,^3^ are projected to increase, indicating that TCs are significant public health concerns. TC exposure is associated with cardiovascular disease (CVD) hospitalizations^7^ and mortality.^8^ For example, Parks *et al*. reported that each additional day of TC exposure is associated with a 1.2% increase in CVD mortality in the United States.^8^

Nonetheless, substantial knowledge gaps persist in our understanding of the association between TC exposure and the risk of developing CVD. First, recent research has rarely addressed developing countries or regions, especially those that are frequently affected by TCs.^1^ A systematic review of 48 studies on the TC–CVD associations revealed that 42 studies were conducted in the United States.^1^ Although mainland China is a global hotspot for TCs, accounting for approximately one-third of worldwide person-day exposure,^4^ research linking TC exposure with CVD in this region remains notably limited. Second, existing studies largely focus on individual TCs within a single location. Most TC-related investigations either focus on examining a single event (e.g. Hurricane Katrina^9^ or Hurricane Sandy^10^) or a single geographic region (e.g. Florida^11^ or Taiwan^12^). Given that TC-related risks vary substantially across individual events and locations,^13^ nationwide research that covers multiple TC events can provide more comprehensive insights for policy formulation. Third, the current findings are inconsistent and often do not account for specific CVD subtypes.^11,14,15^ For example, Burrows *et al*. reported a non-significant association between TC exposure and CVD admissions,^11^ whereas Parks *et al*. reported decreased admissions for valvular heart disease but increased admissions for pulmonary heart disease.^15^ In addition to variations in TC events and regions, these discrepancies may result from differences in the CVD subtypes examined, particularly in terms of their severity and speed of onset. Therefore, clearly defining the CVD subtype of interest is essential.

Ischaemic heart disease (IHD), a subtype of CVD, is the leading cause of premature mortality worldwide.^16^ According to the Global Burden of Disease projections, IHD will remain the primary cause of death globally through 2050, accounting for approximately 186.5 million disability-adjusted life years lost.^17^ Acute coronary syndrome (ACS) encompasses a spectrum of IHD-related disorders, including ST-elevation myocardial infarction (STEMI), non-ST-elevation myocardial infarction (NSTEMI), and unstable angina (UA).^18^ Although ACS is associated with high mortality and poor prognosis,^19^ research on TC–ACS associations remains limited.

Therefore, this study aimed to investigate the short-term association between multiple TC exposures and ACS incidence in China and to explore potential effect modifiers. ACS cases from a nationwide registry in mainland China and TC data from 2015 to 2022 were included to provide novel evidence from the most populous developing country.

## Methods

### Study population

In China, chest pain centres are integrated treatment networks established by hospitals to provide urgent diagnostic and therapeutic services for patients with acute chest pain (more information is provided in Text  *S1*).^20^ In 2015, the Chinese Cardiovascular Association (CCA) launched a nationwide, multicentre chest pain data registry system, namely, the CCA Database–Chest Pain Center.^21–23^ Hospitals with chest pain centres are required to submit patient data, including demographic information, risk factors, comorbidities, admission timelines, clinical diagnoses and treatments, and disease progression or outcomes, to the CCA Database–Chest Pain Center. Detailed variable information is provided in Text  *S2*. Details on data quality control are described in Text  *S3*.

A total of 3 094 812 patients diagnosed with STEMI, NSTEMI, or UA between 1 January 2015, and 31 December 2022, were enrolled in the study through the CCA Database–Chest Pain Center. Onset location and time are crucial factors for TC exposure assessment. Owing to potential information bias in the onset address reported by patients or their families, combined with the more than 50% missing data and the tendency of chest pain patients to choose nearby hospitals with chest pain centres, hospital addresses were used to determine TC exposure and other environmental variables of patients.^24^ Consequently, patients who transferred from external hospitals (*n* = 514 872) and those whose onset times were missing (*n* = 16 160) were excluded because of inaccurate exposure assessment (see Supplementary data online, *Figure S1*). This study was approved by the CCA Database–Chest Pain Center (approval number CCA-CPC-RPA-20240122002). All the data were deidentified, and all the analyses strictly followed the confidentiality protocols established by the CCA.

### Environmental data

The original TC data, with a 3-hour temporal resolution, were obtained from the International Best Track Archive for Climate Stewardship (IBTrACS, version 04r01).^25^ Recognized by the World Meteorological Organization as the most comprehensive global TC dataset,^26^ the IBTrACS integrates information from 12 distinct sources, providing a unified, publicly available best-track dataset that facilitates interagency comparisons.^27^ The raw TC data were processed via the following three steps: (i) from 2015 to 2022, TCs with trajectories passing within a 300-km buffer zone around mainland China, potentially impacting the region, were selected from a total of 878 global TCs; (ii) the central position, pressure, radius, and maximum sustained wind speed of the selected TCs were input into the Willoughby wind field model,^28^ which was implemented through the R package ***StormR***,^29^ to generate grid-level wind fields with an hourly temporal resolution and a spatial resolution of 18.6 km × 18.6 km at the equator; and (iii) hospital locations were mapped onto the wind speed grid to calculate the maximum sustained wind speed on each day from 2015 to 2022 for all hospitals. Additional details are provided in Text  *S4* and Supplementary data online, *Figure S2*. TC exposure was defined as a binary variable, which captures exposure on days when the maximum sustained wind speed is ≥17.5 m/s (63 km/h, 39 mph, or 34 knots).^30^

Data on other environmental factors were also collected as potential covariates. Temperature and relative humidity data, with a daily temporal resolution and a spatial resolution of 0.1° × 0.1° (approximately 11 km × 11 km at the equator), were obtained from the fifth-generation global atmospheric reanalysis dataset of the European Centre for Medium-Range Weather Forecasts, which has demonstrated high accuracy in China.^31^ Air pollutant data, including fine particulate matter (PM_2.5_) and ozone (O_3_) data, with a daily temporal resolution and a spatial resolution of 1 km × 1 km, were sourced from the ChinaHighAirPollutants dataset.^32,33^ As in the TC data processing step, the hospital locations were mapped onto environmental data grids to estimate the daily temperature, relative humidity, PM_2.5_ levels, and O_3_ levels.

### Statistical analyses

In this study, given that TCs are relatively rare, a time-stratified case-crossover design was employed to evaluate the association between TC exposure and the risk of ACS onset by comparing TC days with non-TC days. In accordance with previous research,^15,30^ a conditional quasi-Poisson model was chosen to address potential overdispersion in ACS onset, into which a distributed lag non-linear model (DLNM) was incorporated to capture potential non-linear lagged structures in the TC–ACS association. The Julian day of the year^30^ was matched to account for seasonal variation. The location was matched to account for location-specific baseline risks of ACS, which may result from regional variations in climate, landscape, altitude, ethnicity, diet, and lifestyle habits. For example, if a TC occurred at a given hospital on 15 October 2017, all other data for the same date from 2015 through 2022 at that hospital were considered non-TC days for comparison. The expectation of ACS onset can be obtained as follows:


$$\begin{array}{rl} log[E(Y_{it})] & =crossbasis(TC_{it},lag=14,dflag=3)+temperature_{it}\\ & \;+humidity_{it}+DOW+ns(year,df=3)+location\\ & \;+season+intercept, \end{array}$$

where *i* represents a hospital and *t* represents a specific day. With respect to the crossbasis structure in the DLNM, a lag period of 0–14 days was selected to include enough lag expansion,^30^ a linear function was used for the exposure dimension because of the binary TC variable, and a natural cubic spline (ns) with three degrees of freedom (df) was chosen for the lag dimension. This specification of the DLNM not only preserves the straightforward interpretation of TC as a binary variable in the exposure dimension but also allows for flexible, non-linear patterns in the TC effect in the lag dimension. Meteorological variables, including temperature and humidity, factors associated with TC exposure and incident ACS,^34^ were included as covariates. The day of the week (DOW) and the ns function for the year (with three df) were adjusted to account for intraweek variability and long-term trends. Because the season and location were matched, the conditional quasi-Poisson model does not include the two terms or the intercept. The ***gnm*** and ***dlnm*** packages in R were used to fit the conditional quasi-Poisson model and establish the crossbasis analysis, respectively.

Sensitivity analyses were conducted to evaluate the robustness of the TC–ACS associations with respect to changes in the exposure definition, study population, and statistical model. First, the Boose wind field model,^35^ another widely used method for modelling TC trajectories, was applied to generate exposure data. Second, to account for the possibility of multiple TCs occurring at the same time, wind speed exposure was determined on the basis of data from the nearest weather station (see Text  *S5*). Third, a higher wind speed threshold (33 m/s, 119 km/h, 74 mph, or 64 knots) was employed to classify exposure to hurricanes instead of TCs.^30^ Fourth, to address potential confounding effects stemming from the COVID-19 pandemic, participants with confirmed COVID-19 diagnoses were excluded. Fifth, the analysis was restricted to individuals who provided a self-reported onset address (available only at the county level because of data de-identification) and whose address occurred within the same county as the hospital as these individuals were less likely to experience exposure misclassification. Sixth, air pollutants (including PM_2.5_ and O_3_) were associated with both TC exposure^36,37^ and ACS risk^24^ and were included as potential mediators. Seventh, the distance from the hospital to the coastline (see Text  *S6*) was adjusted, as this variable is associated with both TC exposure and the spatial distribution of ACS cases, potentially serving as a confounder. Finally, meteorological variables were removed from the analysis to estimate the total association rather than the direct association between TC and ACS, as these variables are associated with both TC and ACS and are therefore potential mediators.^34^

Subgroup analyses were conducted to identify susceptible populations. Potential effect modifiers included sex (male and female), age (≥60 and <60 years), education (primary school and below, secondary school, and college and above), ethnic group (Han and minority), distance from the coastline (categorized into tertiles), gross domestic product (GDP) (categorized into tertiles by city-level GDP; Text  *S6*), region (urban and rural), count of risk factors (0, 1–2, and >2), count of comorbidities (0, 1–2, and >2), and disease status (new onset and recurrent; Text  *S7*). Technically, the TC–ACS associations were estimated independently within each subgroup, and the statistical significance of the difference between subgroups was evaluated by 2-sample *z* tests as follows^24^:


$$z=\frac{\left(\beta_{1}-\beta_{2}\right)}{\sqrt{SE_{1}^{2}+SE_{2}^{2}}},$$

where $\beta_{1}$ and $\beta_{2}$ are regression coefficients of TC from the conditional quasi-Poisson model and where $SE_{1}$ and $SE_{2}$ are the corresponding standard errors. A false discovery rate (FDR) correction was applied to the subgroup analyses within each TC–disease association to control for false positive findings.

All the statistical analyses were conducted using R (version 4.2.1). Two-tailed *P* values <.05 were considered to indicate statistical significance. The TC–ACS associations are represented by the relative risk (RR) and 95% confidence interval (CI).

## Results

**Figure 1:**
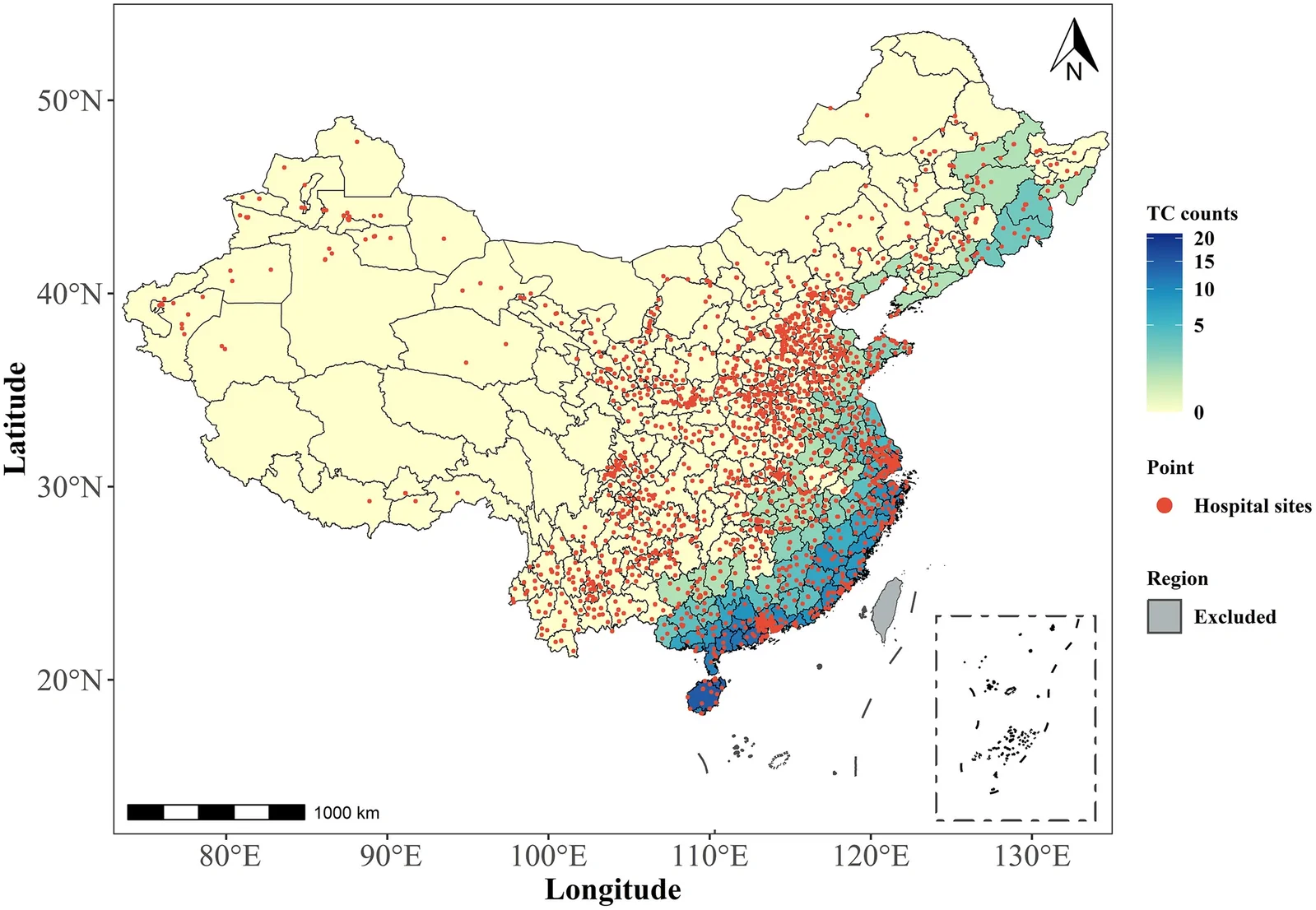
Geographic distribution of chest pain centres and TCs. The figure shows the 2238 hospitals included in the study. The shading of the cities indicates the number of TCs that made landfall in each region, with a total of 126 cities experiencing at least one TC between 2015 and 2022. Abbreviation: TC, tropical cyclone

In this study, 2 563 780 ACS patients from 2238 hospitals across 318 cities in China between 1 January 2015 and 31 December 2022, were included (*Figure 1*). The mean age of the study population was 64.0 years (standard deviation: 12.4 years), and 68% were male, as detailed in *Table 1*. The proportion of males was highest among patients with STEMI, reaching 76%. Patients with NSTEMI presented a greater prevalence of comorbidities, with 10% exhibiting more than two conditions. Following revascularization, significant improvements in thrombolysis in terms of myocardial infarction flow grades were observed across all ACS subtypes (see Supplementary data online, *Table S1*). Compared with those of the population initially recruited from the CCA, the basic demographic characteristics of the final analysed population, such as their age, sex, educational level, and ethnicity, remained largely unchanged (see Supplementary data online, *Table S2*).

**Table 1:** General characteristics of the study population

Characteristic	ACS *n* = 2 563 780	STEMI *n* = 930 378	NSTEMI *n* = 578 083	UA *n* = 1 055 319
Demographic characteristics				
Age, years	64.0 ± 12.4	62.7 ± 13.0	65.0 ± 12.7	64.6 ± 11.6
Male sex	1 743 531 (68)	706 648 (76)	403 303 (70)	633 580 (60)
Education				
Primary school and below	203 011 (44)	73 150 (45)	46 126 (42)	83 735 (44)
Secondary school	223 313 (48)	74 616 (46)	55 950 (51)	92 747 (49)
College and above	36 933 (8)^a^	14 142 (9)	8593 (8)	14 198 (7)
Han ethnic group	1 002 304 (94)	349 365 (94)	239 927 (94)	413 012 (93)
Risk factors				
Hypertension	884 155 (55)	274 400 (48)	222 145 (57)	387 610 (59)
Hyperlipidaemia	325 254 (20)	111 760 (20)	83 617 (22)	129 877 (20)
Diabetes	375 313 (24)	124 427 (22)	101 664 (27)	149 222 (24)
Smoke	450 699 (28)	195 567 (34)	116 695 (30)	138 437 (21)
Obesity	88 948 (6)	34 160 (6)	21 834 (6)	32 954 (5)
Family history of premature CVD	38 315 (2)	16 064 (3)	9192 (2)	13 059 (2)
Count of risk factors ^b^				
0	354 646 (22)	133 024 (24)	75 744 (20)	145 878 (23)
1 or 2	1 011 247 (64)	350 492 (62)	245 580 (64)	415 175 (65)
>2	218 018 (14)	77 793 (14)	60 136 (16)	80 089 (12)
Comorbidities				
Coronary heart disease	786 813 (49)	235 820 (41)	187 861 (49)	363 132 (56)
Atrial fibrillation	68 472 (4)	21 221 (4)	20 605 (5)	26 646 (4)
Chronic heart failure	119 179 (8)	32 968 (6)	35 523 (10)	50 688 (8)
Valvular heart disease	24 852 (2)	6451 (1)	8719 (2)	9682 (1)
Cerebrovascular disease	139 044 (9)	40 514 (7)	36 273 (10)	62 257 (10)
Peripheral arterial disease	68 681 (4)	19 478 (4)	20 023 (5)	29 180 (5)
Aortic aneurysm	2292 (0.1)	592 (0.1)	815 (0.2)	885 (0.1)
Chronic obstructive pulmonary disease	35 384 (2)	10 308 (2)	10 570 (3)	14 506 (2)
Chronic kidney disease	60 206 (4)	17 502 (3)	23 846 (6)	18 858 (3)
Anaemia	48 540 (3)	13 933 (3)	19 122 (5)	15 485 (2)
Peptic ulcer	26 681 (2)	9176 (2)	6948 (2)	10 557 (2)
Thyroid disorders	33 654 (2)	8143 (1)	9416 (3)	16 095 (3)
Cancer	17 406 (1)	6085 (1)	4963 (1)	6358 (1)
Count of comorbidities ^c^				
0	595 411 (39)	242 975 (46)	137 006 (37)	215 430 (34)
1 or 2	817 186 (54)	260 728 (49)	192 283 (52)	364 175 (58)
>2	111 450 (7)	27 129 (5)	37 050 (10)	47 271 (8)

Values are presented as mean (mean ± standard deviations), or *n* (%).

^a^Because of missing data, the percentage reflects the proportion of this category relative to all non-missing values.

^b^The total number of the aforementioned 6 risk factors among the study participants.

^c^The total number of the aforementioned 13 comorbidities among the study participants.

Abbreviations: ACS, acute coronary syndrome; STEMI, ST-elevation myocardial infarction; NSTEMI, non-ST-elevation myocardial infarction; UA, unstable angina; and CVD, cardiovascular disease.

A total of 102 TCs (listed in Supplementary data online, *Table S3*; trajectories shown in Supplementary data online, *Figure S3*) were analysed. During the study period, 126 cities and 840 hospitals experienced at least one TC, with Zhuhai city recording the greatest number of TCs, at 21. Descriptive analyses of environmental factors, including the wind speed, temperature, humidity, and PM_2.5_ and O_3_ concentrations observed on the day of ACS patient admission, are provided in Supplementary data online, *Table S4*, and their correlations are summarized in *Table S5*. The average TC-associated wind speed was 14.3 m/s, with a standard deviation of 6.4 m/s. The wind speeds applied in the main analysis process, as derived from the Willoughby wind field model, were strongly correlated (Spearman *r* = 0.884, *P* < .05) with those derived from the Boose wind field model and moderately correlated (Spearman *r* = 0.406, *P* < .05) with measurements from the nearest weather station.

TC exposure was associated with the admission, treatment, and outcomes of ACS patients (*Table 2*). Compared with non-TC days, on TC days, patients exhibited a higher probability of choosing ambulance admission (12% vs 10%). In terms of time metrics, possibly due to the worse weather conditions on TC days, patients experienced delays in self-referral to the hospital (5.8 h vs 5.3 h) and the time from admission to catheterization room entry (1.0 h vs 0.9 h) on TC days but shorter ambulance transport times (1.8 h vs 2.0 h). Additionally, on TC days, patients exhibited shorter hospital stays, and higher medical costs.

**Table 2:** Influences of TC on admission and treatment of ACS patients

	Cases in non-TC days *n* = 2 561 688	Cases in TC days *n* = 2092
Mode of arrival ^a^		
Ambulance	253 829 (10)	245 (12)
Self-referral	2 240 852 (88)	1760 (84)
In-hospital onset	67 007 (3)	87 (4)
Time metrics (hours)		
For ambulance		
Symptom-to-door time	2.0 [1.0–4.6]	1.8 [1.0–4.0]
Door-to-doctor time	0.0 [0.0–0.0]	0.0 [0.0–0.0]
For self-referral		
Symptom-to-door time	5.3 [1.8–27.9]	5.8 [1.8–34.4]
Door-to-doctor time	0.0 [0.0–0.1]	0.0 [0.0–0.1]
For all		
Consultation time ^b^	0.1 [0.0–0.1]	0.1 [0.0–0.1]
Door-to-cath lab time	0.9 [0.6–1.4]	1.0 [0.7–1.4]
Treatment		
Length of stay (days)	7 [4–10]	6 [4–9]
Hospitalization cost (Yuan)	15 941 [6092–36 052]	24 446 [7720–43 586]
Patient outcomes		
Discharge	2 324 916 (91)	1899 (91)
Transfer to other hospitals	151 881 (6)	105 (5)
Transfer to other departments	24 049 (0.9)	23 (1)
Death	49 616 (2)	50 (2)

Values are presented as *n* (%), or median [interquartile range].

^a^Patients transferred from other hospitals were excluded and are therefore not presented.

^b^The time consumed from the decision to proceed with a cardiology consultation to the completion of the consultation.

Abbreviations: TC, tropical cyclone; ACS, acute coronary syndrome.

**Figure 2:**
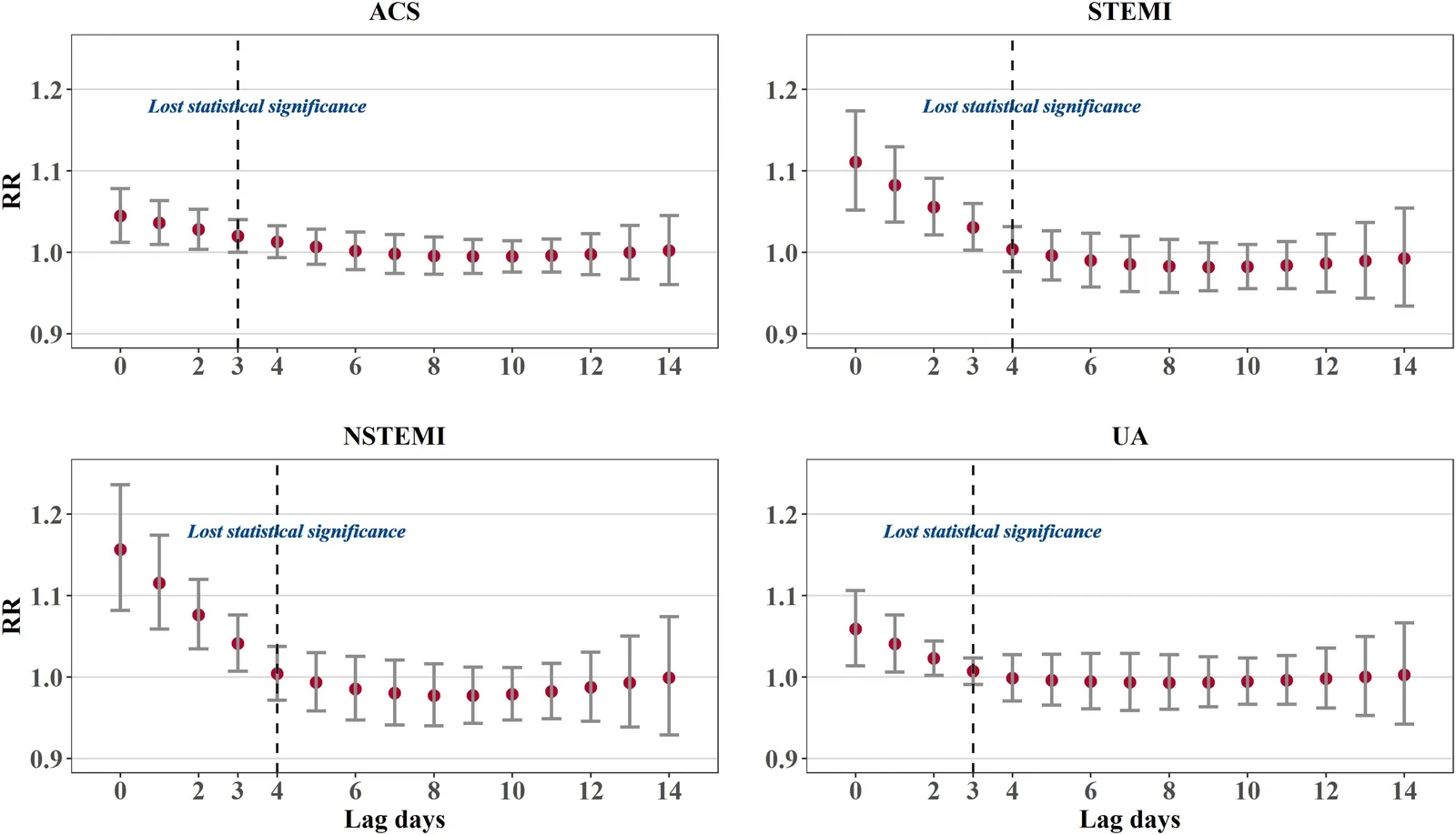
Lag structures of the TC–ACS association. For the red points, the *y*-coordinate denotes the estimated ACS risk on TC days relative to non-TC days. The *x*-coordinate indicates the lag: 0 denotes the TC day, and any non-zero value indicates the number of days after TC. The grey error bars denote the confidence intervals of the effect estimates. The black dashed line indicates the lag at which the association is no longer statistically significant. All the models included crossbasis analysis for TC occurrence, temperature, humidity, day of week, and the natural spline of the year. Abbreviations: ACS, acute coronary syndrome; STEMI, ST-elevation myocardial infarction; NSTEMI, non-ST-elevation myocardial infarction; UA, unstable angina; RR, relative risk; TC, tropical cyclone

TC exposure was positively associated with the risk of developing ACS, STEMI, NSTEMI, and UA (*Figure 2*). These associations peaked on TC days, with RRs (95% CIs) of 1.05 (1.01–1.09), 1.11 (1.06–1.17), 1.16 (1.09–1.24), and 1.06 (1.02–1.10), respectively. The strength of the association decreased over longer lag periods and lost statistical significance after approximately 2–3 days. Consequently, a 0–3-day interval was selected to evaluate the cumulative disease risk associated with TC exposure. Over this interval, TC exposure remained positively associated with the risk of developing ACS, STEMI, NSTEMI, and UA, with RRs (95% CIs) of 1.14 (1.02–1.27), 1.31 (1.13–1.52), 1.45 (1.21–1.73), and 1.14 (1.02–1.26), respectively.

In the sensitivity analyses, modifications to the TC definition, study population, or statistical model did not lead to significant changes in the strength, direction, or statistical significance of the associations of TC exposure with the risk of developing ACS, STEMI, NSTEMI, or UA. For example, when wind speeds were estimated with the Boose model, the associated risks were 1.18 (1.03–1.36), 1.18 (1.01–1.37), 1.24 (1.05–1.47), and 1.13 (1.01–1.25) (see Supplementary data online, *Table S6*). The use of the Boose model yielded associations that differed only slightly from the primary findings, indicating that the results do not depend on the wind field model chosen. The incorporation of wind speed data from meteorological stations did not substantially alter the results (see Supplementary data online, *Table S7*), suggesting that the combined effects of multiple TCs may not need consideration at this stage. Defining exposure on the basis of hurricane criteria produced findings closely resembling the primary results (see Supplementary data online, *Table S8*), suggesting the possible absence of a distinct exposure threshold once the TC criterion is met. The exclusion of COVID-19 cases influenced the results negligibly (see Supplementary data online, *Table S9*), indicating that COVID-19 is unlikely to function as a confounder in TC–ACS associations. Restricting the study population to individuals whose onset and hospitalization occurred in the same county did not render the results non-significant (see Supplementary data online, *Table S10*), indicating that exposure misclassification was limited. Additional adjustment for air pollutants did not markedly attenuate the TC–ACS associations (see Supplementary data online, *Tables S11–12*), suggesting that they are unlikely to serve as potential mediators. Further adjustment for distance from the coastline did not markedly alter the results (see Supplementary data online, *Table S13*), suggesting a low risk of confounding from this factor. In contrast, removing meteorological variables from the model increased the strength of the TC–ACS associations (see Supplementary data online, *Table S14*), suggesting that these variables may partially mediate the observed association.

The associations of TC exposure with the risk of developing both ACS and NSTEMI were stronger in males than in females (both *P-adj* = .015). Compared with those with at least a college-level education, individuals with primary-level schooling or below exhibited a stronger TC–UA association (*P-adj* = .005). Individuals with at least three risk factors demonstrated stronger TC–ACS (*P-adj* = .244), TC–NSTEMI (*P-adj* = .045), and TC–UA (*P-adj* = .125) associations than those without any risk factors did, with the TC–UA association not being statistically significant after FDR adjustment. There was no evidence that the region, number of comorbidities or disease status modified the TC–ACS association. The TC–ACS association appeared stronger among cases near the coastline and cases in locations with higher GDP levels; however, these differences lost statistical significance after FDR adjustment. Details are provided in *Table 3*.

**Table 3:** ACS onset risk associated with TC over lags of 0 to 3 days as stratified by different subgroups

Group	ACS		STEMI		NSTEMI		UA	
	RR (95% CI)	*P* ^a^ (*P*_adj_ ^b^)	RR (95% CI)	*P* (*P*_adj_)	RR (95% CI)	*P* (*P*_adj_)	RR (95% CI)	*P* (*P*_adj_)
Age, years								
<60	1.29 (1.11–1.50)	Ref	1.50 (1.19–1.89)	Ref	1.51 (1.18–1.94)	Ref	1.18 (0.95–1.48)	Ref
≥60	1.11 (0.98–1.25)	0.128 (0.384)	1.19 (0.99–1.43)	0.121 (0.997)	1.44 (1.16–1.79)	0.776 (0.905)	1.07 (0.96–1.20)	0.423 (0.635)
Sex								
Male	1.29 (1.12–1.49)	Ref	1.35 (1.13–1.61)	Ref	1.59 (1.22–2.08)	Ref	1.11 (0.94–1.30)	Ref
Female	0.99 (0.94–1.05)	**0.001** (**0.015)**	1.17 (0.92–1.50)	0.365 (0.997)	0.90 (0.72–1.11)	**0.001** (**0.015)**	1.09 (0.95–1.27)	0.897 (0.897)
Education								
Primary school and below	1.74 (0.74–4.08)	Ref	1.56 (0.69–3.50)	Ref	1.99 (1.33–2.97)	Ref	1.81 (1.29–2.54)	Ref
Secondary school	1.66 (0.68–4.03)	0.938 (0.938)	1.65 (0.66–4.12)	0.928 (0.997)	1.88 (1.30–2.70)	0.835 (0.905)	1.52 (0.70–3.31)	0.681 (0.786)
College and above	1.58 (1.24–2.02)	0.834 (0.938)	1.73 (1.31–2.29)	0.809 (0.997)	1.79 (1.32–2.42)	0.679 (0.905)	0.94 (0.83–1.06)	**<0.001** (**0.005)**
Ethnic group								
Han	1.50 (1.19–1.90)	Ref	1.59 (0.71–3.58)	Ref	1.72 (1.25–2.37)	Ref	1.29 (1.06–1.56)	Ref
Minority	0.94 (0.37–2.42)	0.347 (0.868)	1.62 (0.69–3.78)	0.979 (0.997)	0.81 (0.57–1.15)	**0.002** (**0.015)**	0.93 (0.57–1.52)	0.228 (0.570)
Distance from the coastline								
Near	1.51 (1.19–1.91)	Ref	1.50 (1.10–2.05)	Ref	1.61 (1.23–2.10)	Ref	1.53 (1.16–2.03)	Ref
Middle	1.38 (1.09–1.75)	0.604 (0.938)	1.50 (1.12–2.02)	0.997 (0.997)	1.52 (1.11–2.08)	0.790 (0.905)	1.34 (0.89–2.01)	0.596 (0.745)
Far	1.14 (0.98–1.32)	**0.046** (0.230)	1.36 (1.11–1.66)	0.591 (0.997)	1.44 (1.12–1.85)	0.556 (0.905)	1.06 (0.96–1.19)	**0.018** (0.125)
GDP level								
Low	1.20 (1.01–1.43)	Ref	1.35 (1.05–1.72)	Ref	1.42 (1.07–1.87)	Ref	1.20 (0.92–1.57)	Ref
Middle	1.24 (1.03–1.48)	0.817 (0.938)	1.53 (1.23–1.90)	0.443 (0.997)	1.47 (1.12–1.94)	0.845 (0.905)	1.05 (0.96–1.16)	0.371 (0.625)
High	1.72 (1.27–2.34)	**0.046** (0.230)	1.68 (1.23–2.28)	0.273 (0.997)	1.96 (1.33–2.88)	0.181 (0.679)	1.76 (1.23–2.51)	0.095 (0.285)
Region								
Urban	1.21 (1.04–1.41)	Ref	1.22 (0.99–1.50)	Ref	1.51 (1.18–1.93)	Ref	1.26 (1.00–1.59)	Ref
Rural	1.15 (0.97–1.36)	0.625 (0.938)	1.49 (1.18–1.89)	0.208 (0.997)	1.44 (1.12–1.86)	0.803 (0.905)	0.97 (0.80–1.17)	0.073 (0.274)
Count of risk factors								
0	1.71 (1.42–2.04)	Ref	1.55 (1.24–1.94)	Ref	2.13 (1.67–2.72)	Ref	1.55 (1.18–2.05)	Ref
1 to 2	1.82 (1.56–2.12)	0.595 (0.938)	1.61 (0.65–4.04)	0.937 (0.997)	2.42 (1.98–2.95)	0.435 (0.905)	1.82 (1.47–2.25)	0.375 (0.625)
>2	2.20 (1.80–2.69)	0.065 (0.244)	1.52 (1.22–1.91)	0.901 (0.997)	3.42 (2.65–4.42)	**0.009** (**0.045)**	2.39 (1.85–3.09)	**0.025** (0.125)
Count of comorbidities								
0	1.60 (1.22–2.09)	Ref	1.62 (0.64–4.07)	Ref	1.67 (1.25–2.24)	Ref	1.52 (0.68–3.40)	Ref
1 to 2	1.63 (1.24–2.15)	0.918 (0.938)	1.61 (1.18–2.20)	0.990 (0.997)	1.77 (1.29–2.42)	0.795 (0.905)	1.63 (1.08–2.46)	0.877 (0.897)
>2	1.53 (1.20–1.95)	0.803 (0.938)	1.03 (0.98–1.08)	0.336 (0.997)	1.64 (1.23–2.19)	0.944 (0.944)	1.16 (0.92–1.45)	0.525 (0.716)
Disease status								
New-onset	1.30 (1.16–1.46)	Ref	1.28 (1.16–1.58)	Ref	1.36 (1.14–1.63)	Ref	1.21 (1.01–1.45)	Ref
Recurrent	1.41 (1.03–1.93)	0.652 (0.938)	1.40 (0.95–2.06)	0.685 (0.997)	1.68 (1.16–2.43)	0.323 (0.905)	1.47 (1.04–2.07)	0.328 (0.625)

^a^ *P* denotes the tests for differences across subgroups.

^b^ *P*_adj_ indicates *P*-values adjusted using false discovery rate correction. Treating the associations of TC with the four outcomes as separate groups, the *P*-values were adjusted within each group.

All the models included crossbasis analysis for TC occurrence, temperature, humidity, day of week, and the natural spline of the year.

Abbreviations: ACS, acute coronary syndrome; TC, tropical cyclone; STEMI, ST-elevation myocardial infarction; NSTEMI, non-ST-elevation myocardial infarction; UA, unstable angina; RR, relative risk; CI, confidence interval; *P*-int, *P* for interaction; Ref, reference group; GDP, gross domestic product; CVD, cardiovascular disease.

## Discussion

This nationwide study demonstrated that short-term exposure to TCs was associated with an increased risk of developing ACS and its subtypes on the basis of an analysis of data from 2.56 million ACS patients and 102 TCs in mainland China from 2015 to 2022 (*Structured Graphical Abstract*). These associations peaked on TC days and lost statistical significance after approximately 2–3 days. Stronger associations were observed among males, individuals with lower education levels, and those with more ACS risk factors. To our knowledge, this is the first study to focus on investigating the associations of multiple TC exposures with incident ACS. Our findings provide robust evidence from China, which is the most populous developing country, highlighting the importance of reducing the TC-associated ACS burden and prioritizing the protection of vulnerable populations.

### Comparison with other studies

Previous studies have investigated TC–CVD associations, yet most investigations have focused on individual TC events.^9,10,38–41^ For example, Kim *et al*. reported a 6% increase in CVD-related mortality among older adults in New Jersey during the month following Hurricane Sandy.^39^ However, the conclusions drawn from the occurrence of a single TC may be influenced by its particular landfall time and wind speed, thereby limiting its generalizability to other TCs.^13^ To date, five investigations have explored the relationship between exposure to multiple TCs and incident CVD, but their findings are inconsistent.^7,8,11,13,15^ For example, in a 12-year analysis of TC exposure across the United States, Yan *et al*. reported a 3% increase in CVD hospitalization rates during TC exposure periods compared with non-exposure intervals.^7^ In contrast, in their 18-year study of TCs in Florida, Burrows *et al*. did not observe statistically significant findings.^11^ Additionally, while Parks *et al*. similarly reported a non-significant association between TC exposure and overall CVD risk, they reported heterogeneity among different CVD subtypes.^8^ Specifically, TC exposure was associated with increased admissions for ACS but decreased admissions for valvular heart disease. These findings may be attributable to disease severity, as limited transportation and medical resources are prioritized to ensure the admission and treatment of serious conditions, such as ACS, during the TC period, thus highlighting the importance of examining CVD subtypes in related research.^15^ In light of these research gaps, the associations between exposure to multiple TCs and the risks of developing the most burdensome CVD subtypes, specifically ACS, were investigated in this study. By employing a large-scale dataset and an extended study period, this study provides novel evidence from a developing country, thereby helping to address disparities in environmental health.

Owing to a lack of individual-level data, limited studies have explored effect modifiers in the TC–CVD associations. Researchers have investigated effect modifiers by using community-level data to determine socioeconomic status^8,11^ or by leveraging medical data, such as hospital visit records^10^ or Medicare datasets,^11^ to obtain individual demographic information. It has been shown that females,^10^ individuals living in severe poverty,^11^ and those with significant social vulnerability^8^ are at increased risk of developing CVD following TC exposure. This study revealed that males and individuals with lower education levels exhibit greater risks of developing ACS following TC exposure. Educational level, as a proxy for income, aligns with findings from previous research. Differences in the effect modification by sex compared with existing studies may be attributed to variations in population characteristics or TC intensity. Notably, for this research, individual-level CVD risk factor and comorbidity data were obtained, and, for the first time, CVD risk factors were identified as effect modifiers, thereby offering valuable guidance for future clinical and public health efforts to mitigate the burden of TC-related ACS. Moreover, the results of this study revealed that TC–ACS associations differed between new-onset and recurrent cases, although the difference was not statistically significant. Despite the relatively small number of recurrent cases in this study, our results suggest that patients with preexisting ACS may represent a population that is vulnerable to TC, which should be researched further to provide additional evidence.

### Potential mechanisms

Although the precise mechanisms remain unclear, TC-associated stress may partially clarify the TC–ACS associations. TCs may elicit acute psychological stress,^42^ increasing inflammation and plaque vulnerability,^43^ and ultimately precipitating ACS^44^; this hypothesis accounts for the maximum increase in ACS risk observed on TC days. Following TC exposure, sustained chronic stress^45^ can activate the hypothalamic–pituitary–adrenocortical and sympatho-adrenomedullary axes,^42^ leading to catecholamine release and accumulation^46^ and autonomic dysfunction,^47^ all of which are linked to hypertension and cardiovascular events,^48,49^ thereby explaining the lagged association between TCs and ACS. On the one hand, stress-sensitive populations, such as those living in poverty,^50,51^ may experience elevated TC-related ACS risks. However, a stronger (although not significant after FDR correction) TC–ACS association was identified at locations near the coastline and among those with higher GDP levels in this study. Considering the actual economic development situation in China, it was hypothesized that because the GDP is closely correlated with the distance from the coastline (Spearman *r* = −0.371, *P* < .05), it may not accurately reflect the individual income status of patients. While income data were unavailable, lower educational attainment (a proxy for income) was associated with higher risk. On the other hand, individuals with existing plaques or multiple ACS risk factors could be more susceptible to TC exposure,^43^ which is consistent with our subgroup analyses. Moreover, changes in environmental conditions during power failures, including suboptimal indoor temperatures^52^ and emissions from portable generators^53^ and the rapid proliferation of mould bioaerosols,^54^ may further increase the risk of ACS.

### Clinical and public health implications

This study revealed that TCs were associated with an approximately 14% increase in ACS risk within 3 days and that TCs may delay hospital admission and subsequent treatment. In the present study, ACS occurs most often between October and December, a pattern consistent with previous reports,^55,56^ whereas TCs are most common from July to September, indicating a temporal mismatch during their peak periods. On the one hand, this pattern may be considered favourable because the baseline summer burden of ACS is relatively low; consequently, the additional number of TC-attributable cases is also relatively small. However, this finding poses specific challenges for policymaking and highlights the need to routinely strengthen ACS-related medical resource reserves in summer in regions that are frequently affected by TCs and serve large catchment areas. Although the absolute increase in risk is modest, the substantial TC burden in China and the wide geographic footprint of individual events suggest a meaningful number of excess ACS cases. These additional cases may strain chest pain centres, especially given the expectation of more frequent and intense TCs in the future. For example, when Super Typhoon Maysak made landfall in Heilongjiang on 3 September 2020, 17 ACS patients were admitted to a hospital in Harbin on that day, compared with 5 on the previous day. This burden would likely have been even greater if other conditions requiring admission to chest pain centres were included. TCs may also disrupt transportation and delay admission, which is associated with increased mortality among ACS patients.^57^ In addition, TC-induced stress and psychosocial responses can broadly affect workers in healthcare, transportation, and related sectors, further impeding timely care. Collectively, these findings indicate that TC-related ACS risk is not merely a clinical issue but also a significant and complex public health concern that warrants seasonally tailored preparedness and resource allocation.

Reducing the burden of TC-associated ACS requires collaboration among governments, the public, and healthcare institutions. Given the seasonality of TCs, the recommendations proposed in this study apply primarily to the summer period in China, when TCs are most prevalent. When these recommendations are generalized, particular attention should be given to differences in the seasonal pattern of TC occurrence. Governments should increase ambulance reserves to respond to surges in patient transfer demand on TC days and to reduce transfer times; expand medical resources and increase capacity (e.g. emergency rooms, coronary care unit beds, and catheterization laboratory capacity) at key or designated cardiovascular care facilities in regions with frequent TC and appropriately direct patients to these priority sites; establish mobile cardiac monitoring units to expand coverage in remote areas and alleviate the burden on chest pain centres during surge periods; and refine community-based disaster preparedness programs to enhance public education on disaster awareness and to facilitate the management of vulnerable populations. The public should stay informed regarding TC updates, learn about disaster prevention and mitigation strategies, stock essential supplies (including medications for chronic conditions), seek prompt medical attention when chest pain symptoms occur, and use ambulance services rather than self-referral to the hospital whenever possible. The management teams of healthcare institutions should maintain sufficient energy reserves to manage potential shortages during TCs, allocate adequate medical resources to handle surges in ACS cases during such events, develop emergency response plans to reduce the time from patient admission to catheterization room access during TC periods, and establish one-to-one telemedicine protocols between hospitals in TC-prone areas and inland hospitals to mitigate medical resource shortages during TC periods.

### Strengths and limitations

This study exhibits several strengths. First, this is the first study to investigate the associations between multiple TC exposures and incident ACS. Through analysis of data from 2.56 million individuals with ACS, robust evidence of TC–ACS associations in a developing country was provided. Second, a wind-field model was used to assess individual-level, high-resolution TC exposure accurately. In addition, the influences of the data sources and model selection on the results were examined, and the conclusions remained consistent. Third, a time-stratified case-crossover design was implemented to account for both geographic location and seasonal variations. This approach helps control for potential unobserved time-space-dependent confounders, such as the seasonality of TC and ACS events, as well as location-specific baseline incidence rates of ACS. This methodological choice strengthens the basis for causal inference. Fourth, a range of potential effect modifiers was explored, highlighting a novel examination of how ACS risk factors and disease history may modify TC–ACS associations.

Several limitations should be acknowledged. First, exposure misclassification may still occur. The use of the hospital address as a proxy for the actual onset location could introduce inaccuracies in exposure assessment. However, the influence of this issue is limited because (i) regions prone to TCs exhibit a high density of chest pain centres, and patients are generally transferred to the nearest facility^24^; (ii) the spatial resolution of the wind field data is 18.6 km × 18.6 km, and evidence suggests that the actual onset location is typically located within a few kilometres of the chest pain centre,^24^ making it highly probable that they share the same wind field grid; (iii) exposure misclassification is non-differential across individuals,^15^ causing any resulting bias to tend towards null^58^; (iv) our sensitivity analysis revealed that restricting the dataset to cases with self-reported onset addresses in the same county as the hospital did not materially change the results, alleviating our concerns about using hospital location, particularly given that TC wind fields are typically larger than a county^15,59^; and (v) rural regions have a lower density of chest pain centres, requiring patients to travel longer distances; nonetheless, our subgroup analysis revealed no significant differences in TC–ACS associations between rural and urban regions, reducing our concerns regarding region-specific exposure misclassification. Second, residual confounding may still occur. Although this study accounted for potential confounders, such as geographic location, month, season, long-term trends, weekdays, temperature and humidity, COVID, distance from the coastline, and air pollutants, any residual confounder must be associated with both TC exposure and incident ACS, independent of these considered factors.^15^ Therefore, while the possibility of residual confounding cannot be excluded completely, its impact on our findings is considered likely to be limited. Third, in the subgroup analyses, the presence of missing data for the stratification variables led to a reduced sample size. This reduction consequently decreased the statistical power and introduced a potential selection bias. Therefore, further investigation into the differences between these subgroups is warranted. Fourth, because some ACS patients may be admitted to hospitals lacking chest pain centre certification or those without chest pain centres, these findings may not be generalizable to all ACS patients in China.

## Conclusions

In conclusion, TC exposure was associated with an increased risk of developing ACS and its subtypes, particularly within the first 0–3 lag days after TC exposure. Males, individuals with lower education levels, and those with more ACS risk factors were at greater risk. Future research priorities include examining other CVD subtypes, extending studies to other TC-prone developing countries, and exploring underlying mechanisms.

## Supplementary Material

ehag066_Supplementary_Data

## Declarations

### Data availability

Anonymized data will be available through a formal application process that will be reviewed by the Data Management Committee of the CCA Database-Chest Pain Center.

## References

[1] Ghosh AK, Demetres MR, Geisler BP, Ssebyala SN, Yang T, Shapiro MF, et al Impact of hurricanes and associated extreme weather events on cardiovascular health: a scoping review. Environ Health Perspect 2022;130:116003. 10.1289/ehp11252 36448792 PMC9710380

[2] Gibbens S . *Hurricane Sandy, Explained*. https://www.nationalgeographic.com/environment/article/hurricane-sandy (12 February 2025, date last accessed).

[3] Young R, Hsiang S. Mortality caused by tropical cyclones in the United States. Nature 2024;635:121–8. 10.1038/s41586-024-07945-5 39358513 PMC11541193

[4] Jing R, Heft-Neal S, Chavas DR, Griswold M, Wang Z, Clark-Ginsberg A, et al Global population profile of tropical cyclone exposure from 2002 to 2019. Nature 2024;626:549–54. 10.1038/s41586-023-06963-z 38122822 PMC12057795

[5] Murakami H, Levin E, Delworth TL, Gudgel R, Hsu PC. Dominant effect of relative tropical Atlantic warming on major hurricane occurrence. Science (New York, N.Y.) 2018;362:794–9. 10.1126/science.aat6711 30262635

[6] Sobel AH, Camargo SJ, Hall TM, Lee CY, Tippett MK, Wing AA. Human influence on tropical cyclone intensity. Science 2016;353:242–6. 10.1126/science.aaf6574 27418502

[7] Yan M, Wilson A, Dominici F, Wang Y, Al-Hamdan M, Crosson W, et al Tropical cyclone exposures and risks of emergency medicare hospital admission for cardiorespiratory diseases in 175 urban United States counties, 1999–2010. Epidemiology 2021;32:315–26. 10.1097/ede.0000000000001337 33591048 PMC8887827

[8] Parks RM, Benavides J, Anderson GB, Nethery RC, Navas-Acien A, Dominici F, et al Association of tropical cyclones with county-level mortality in the US. JAMA 2022;327:946–55. 10.1001/jama.2022.1682 35258534 PMC8905400

[9] Lenane Z, Peacock E, Joyce C, Frohlich ED, Re RN, Muntner P, et al Association of post-traumatic stress disorder symptoms following Hurricane Katrina with incident cardiovascular disease events among older adults with hypertension. Am J Geriatr Psychiatry 2019;27:310–21. 10.1016/j.jagp.2018.11.006 30581139 PMC6476543

[10] Lawrence WR, Lin Z, Lipton EA, Birkhead G, Primeau M, Dong GH, et al After the storm: short-term and long-term health effects following superstorm sandy among the elderly. Disaster Med Public Health Prep 2019;13:28–32. 10.1017/dmp.2018.152 30841951 PMC12150664

[11] Burrows K, Anderson GB, Yan M, Wilson A, Sabath MB, Son JY, et al Health disparities among older adults following tropical cyclone exposure in Florida. Nat Commun 2023;14:2221. 10.1038/s41467-023-37675-7 37076480 PMC10115860

[12] Chang C-M, Chao T-YS, Huang Y-T, Tu Y-F, Sung T-C, Wang J-D, et al Maintaining quality of care among dialysis patients in affected areas after Typhoon Morakot. Int J Environ Res Public Health 2021;18:7400. 10.3390/ijerph18147400 34299851 PMC8305479

[13] Huang W, Yang Z, Zhang Y, Vogt T, Armstrong B, Yu W, et al Tropical cyclone-specific mortality risks and the periods of concern: a multicountry time-series study. PLoS Med 2024;21:e1004341. 10.1371/journal.pmed.1004341 38252630 PMC10843109

[14] Huang W, Gao Y, Xu R, Yang Z, Yu P, Ye T, et al Health effects of cyclones: a systematic review and meta-analysis of epidemiological studies. Environ Health Perspect 2023;131:86001. 10.1289/ehp12158 37639476 PMC10461789

[15] Parks RM, Anderson GB, Nethery RC, Navas-Acien A, Dominici F, Kioumourtzoglou MA. Tropical cyclone exposure is associated with increased hospitalization rates in older adults. Nat Commun 2021;12:1545. 10.1038/s41467-021-21777-1 33750775 PMC7943804

[16] GBD 2021 Diseases and Injuries Collaborators . Global burden of 288 causes of death and life expectancy decomposition in 204 countries and territories and 811 subnational locations, 1990–2021: a systematic analysis for the Global Burden of Disease Study 2021. Lancet 2024;403:2100–32. 10.1016/S0140-6736(24)00367-2 38582094 PMC11126520

[17] GBD 2021 Diseases and Injuries Collaborators . Burden of disease scenarios for 204 countries and territories, 2022–2050: a forecasting analysis for the Global Burden of Disease Study 2021. Lancet 2024;403:2204–56. 10.1016/S0140-6736(24)00685-8 38762325 PMC11121021

[18] Thygesen K, Alpert JS, Jaffe AS, Chaitman BR, Bax JJ, Morrow DA, et al Fourth Universal Definition of Myocardial Infarction (2018). Circulation 2018;138:e618–51. 10.1161/CIR.0000000000000617 30571511

[19] Amsterdam EA, Wenger NK, Brindis RG, Casey DE, Ganiats TG, Holmes DR, et al 2014 AHA/ACC guideline for the management of patients with non-ST-elevation acute coronary syndromes: a report of the American College of Cardiology/American Heart Association Task Force on Practice Guidelines. Circulation 2014;130:e344–426. 10.1161/CIR.0000000000000134 25249585

[20] Zhang Y, Huo Y. Early reperfusion strategy for acute myocardial infarction: a need for clinical implementation. J Zhejiang Univ Sci B 2011;12:629–32. 10.1631/jzus.B1101010 21796802 PMC3150715

[21] Xie Z, Xu Y, Song Y, Wang Y, Han X, Sun A, et al De novo heart failure in patients hospitalized with ST-segment elevation myocardial infarction in contemporary China. Cardiol Plus 2025;10:10–22. 10.1097/cp9.0000000000000108

[22] Wu J, Yang H, Li H, Meng W, Huo Y, Zhang Yi. Distribution of diseases among emergency Chest Pain Center patients from 2018 to 2022: a retrospective analysis from the Chinese Cardiovascular Association Database. Cardiol Plus 2025;10:261–268. 10.1097/cp9.0000000000000139

[23] Zhao L, Geru A, Sun B, Li P, Wang Z, Li L, et al Sex differences in STEMI management and outcomes: a retrospective analysis from the China Chest Pain Center Database. Cardiol Plus 2024;9:159–67. 10.1097/CP9.0000000000000095

[24] Chen R, Jiang Y, Hu J, Chen H, Li H, Meng X, et al Hourly air pollutants and acute coronary syndrome onset in 1.29 million patients. Circulation 2022;145:1749–60. 10.1161/CIRCULATIONAHA.121.057179 35450432

[25] Knapp KR, Kruk MC, Levinson DH, Diamond HJ, Neumann CJ. The International Best Track Archive for Climate Stewardship (IBTrACS): unifying tropical cyclone data. Bull Am Meteorol Soc 2010;91:363–76. 10.1175/2009BAMS2755.1

[26] Schreck, Carl & National Center for Atmospheric Research Staff (Eds). *The Climate Data Guide: IBTrACS: Tropical Cyclone Best Track Data*. https://climatedataguide.ucar.edu/climate-data/ibtracs-tropical-cyclone-best-track-data (27 November 2024, date last accessed).

[27] Gahtan J, Knapp KR, Schreck CJ, Diamond HJ, Kossin JP, Kruk MC. 2024. International Best Track Archive for Climate Stewardship (IBTrACS) Project, Version 4r01. IBTrACS.since1980.v04r01. NOAA National Centers for Environmental Information. (November 27 2024, date last accessed). 10.25921/82ty-9e16

[28] Willoughby HE, Darling RWR, Rahn ME. Parametric representation of the primary hurricane vortex. Part II: a new family of sectionally continuous profiles. Mon Weather Rev 2006;134:1102–20. 10.1175/MWR3106.1

[29] UMR-AMAP . *StormR*. https://github.com/umr-amap/StormR (27 November 2024, date last accessed).

[30] Huang W, Li S, Vogt T, Xu R, Tong S, Molina T, et al Global short-term mortality risk and burden associated with tropical cyclones from 1980 to 2019: a multi-country time-series study. Lancet Planet Health 2023;7:e694–705. 10.1016/s2542-5196(23)00143-2 37558350

[31] Sun Y, Yang F, Liu M, Li Z, Gong X, Wang Y. Evaluation of the weighted mean temperature over China using multiple reanalysis data and radiosonde. Atmos Res 2023;285:106664. 10.1016/j.atmosres.2023.106664

[32] Wei J, Li Z, Lyapustin A, Sun L, Peng Y, Xue W, et al Reconstructing 1-km-resolution high-quality PM2.5 data records from 2000 to 2018 in China: spatiotemporal variations and policy implications. Remote Sens Environ 2021;252:112136. 10.1016/j.rse.2020.112136

[33] Wei J, Li Z, Li K, Dickerson RR, Pinker RT, Wang J, et al Full-coverage mapping and spatiotemporal variations of ground-level ozone (O3) pollution from 2013 to 2020 across China. Remote Sens Environ 2022;270:112775. 10.1016/j.rse.2021.112775

[34] Ezekowitz JA, Bakal JA, Westerhout CM, Giugliano RP, White H, Keltai M, et al The relationship between meteorological conditions and index acute coronary events in a global clinical trial. Int J Cardiol 2013;168:2315–21. 10.1016/j.ijcard.2013.01.061 23416014

[35] Boose ER, Serrano MI, Foster DR. Landscape and regional impacts of Hurricanes in Puerto Rico. Ecol Monogr 2004;74:335–52. 10.1890/02-4057

[36] Shao M, Yang J, Wang J, Chen P, Liu B, Dai Q. Co-occurrence of surface O3, PM2.5 pollution, and tropical cyclones in China. J Geophys Res Atmos 2022;127:e2021JD036310. 10.1029/2021JD036310

[37] Lee YC, Calori G, Hills P, Carmichael GR. Ozone episodes in urban Hong Kong 1994–1999. Atmos Environ 2002;36:1957–68. 10.1016/S1352-2310(02)00150-4

[38] Swerdel JN, Janevic TM, Cosgrove NM, Kostis JB. The effect of Hurricane Sandy on cardiovascular events in New Jersey. J Am Heart Assoc 2014;3:e001354. 10.1161/jaha.114.001354 25488295 PMC4338729

[39] Kim S, Kulkarni PA, Rajan M, Thomas P, Tsai S, Tan C, et al Hurricane Sandy (New Jersey): mortality rates in the following month and quarter. Am J Public Health 2017;107:1304–7. 10.2105/ajph.2017.303826 28640678 PMC5508144

[40] Deere B, Moscona J, Srivastav S, Zimmerman M, Mallya S, Razavi M, et al Incidence of acute myocardial infarction and Hurricane Katrina: four-fold increase eleven years after the storm. J Am Coll Cardiol 2018;71:A1903. 10.1016/S0735-1097(18)32444-6

[41] Becquart NA, Naumova EN, Singh G, Chui KKH. Cardiovascular disease hospitalizations in Louisiana parishes’ elderly before, during and after Hurricane Katrina. Int J Environ Res Public Health 2018;16:74. 10.3390/ijerph16010074 30597886 PMC6339087

[42] Steptoe A, Kivimäki M. Stress and cardiovascular disease. Nat Rev Cardiol 2012;9:360–70. 10.1038/nrcardio.2012.45 22473079

[43] Hinterdobler J, Schott S, Jin H, Meesmann A, Steinsiek A-L, Zimmermann A-S, et al Acute mental stress drives vascular inflammation and promotes plaque destabilization in mouse atherosclerosis. Eur Heart J 2021;42:4077–88. 10.1093/eurheartj/ehab371 34279021 PMC8516477

[44] Shah Prediman K . Mechanisms of plaque vulnerability and rupture. J Am Coll Cardiol 2003;41:S15–22. 10.1016/S0735-1097(02)02834-6 12644336

[45] Tasdik Hasan M, Adhikary G, Mahmood S, Papri N, Shihab HM, Kasujja R, et al Exploring mental health needs and services among affected population in a cyclone affected area in costal Bangladesh: a qualitative case study. Int J Ment Health Syst 2020;14:12. 10.1186/s13033-020-00351-0 32165918 PMC7059662

[46] Ulrich-Lai YM, Herman JP. Neural regulation of endocrine and autonomic stress responses. Nat Rev Neurosci 2009;10:397–409. 10.1038/nrn2647 19469025 PMC4240627

[47] Schneider M, Schwerdtfeger A. Autonomic dysfunction in posttraumatic stress disorder indexed by heart rate variability: a meta-analysis. Psychol Med 2020;50:1937–48. 10.1017/s003329172000207x 32854795 PMC7525781

[48] Grassi G, Mark A, Esler M. The sympathetic nervous system alterations in human hypertension. Circ Res 2015;116:976–90. 10.1161/CIRCRESAHA.116.303604 25767284 PMC4367954

[49] Hillebrand S, Gast KB, de Mutsert R, Swenne CA, Jukema JW, Middeldorp S, et al Heart rate variability and first cardiovascular event in populations without known cardiovascular disease: meta-analysis and dose–response meta-regression. EP Europace 2013;15:742–9. 10.1093/europace/eus341 23370966

[50] Strike PC, Perkins-Porras L, Whitehead DL, McEwan J, Steptoe A. Triggering of acute coronary syndromes by physical exertion and anger: clinical and sociodemographic characteristics. Heart 2006;92:1035–40. 10.1136/hrt.2005.077362 16399852 PMC1861076

[51] Edmondson D, Newman JD, Whang W, Davidson KW. Emotional triggers in myocardial infarction: do they matter? Eur Heart J 2013;34:300–6. 10.1093/eurheartj/ehs398 23178642 PMC3549526

[52] Shultz James M, Galea S. Preparing for the Next Harvey, Irma, or Maria—addressing research gaps. N Engl J Med 2017;377:1804–6. 10.1056/NEJMp1712854 29019711

[53] Lane K, Charles-Guzman K, Wheeler K, Abid Z, Graber N, Matte T. Health effects of coastal storms and flooding in urban areas: a review and vulnerability assessment. J Environ Public Health 2013;2013:913064. 10.1155/2013/913064 23818911 PMC3683478

[54] Schmidlin TW . Public health consequences of the 2008 Hurricane Ike windstorm in Ohio, USA. Nat Hazards 2011;58:235–49. 10.1007/s11069-010-9663-x

[55] Faramand Z, Frisch SO, Martin-Gill C, Landis P, Alrawashdeh M, Al-Robaidi KA, et al Diurnal, weekly and seasonal variations of chest pain in patients transported by emergency medical services. Emerg Med J 2019;36:601–7. 10.1136/emermed-2019-208529 31366626 PMC7316529

[56] Li Y, Du T, Lewin MR, Wang H, Ji X, Zhang Y, et al The seasonality of acute coronary syndrome and its relations with climatic parameters. Am J Emerg Med 2011;29:768–74. 10.1016/j.ajem.2010.02.027 20825896

[57] Xu Q, Liu Y, Li H, Lu Z, Wang M, Huang L, et al Factors of emergency medical services call delay and its impact on prognosis in STEMI patients: findings from the CCA database-chest pain center registry. Int J Cardiol 2025;443:133944. 10.1016/j.ijcard.2025.133944 41043718

[58] Carroll RJ, Ruppert D, Stefanski LA, Crainiceanu CM. Measurement Error in Nonlinear Models: A Modern Perspective, Second Edition (2nd ed.). Chapman and Hall/CRC, 2006. 10.1201/9781420010138

[59] Anderson GB, Ferreri J, Al-Hamdan M, Crosson W, Schumacher A, Guikema S, et al Assessing United States county-level exposure for research on tropical cyclones and human health. Environ Health Perspect 2020;128:107009. 10.1289/EHP6976 33112191 PMC7592507

