# Supplementary material for: Tropical cyclones and acute coronary syndromes: a Chinese nationwide study
Source: Eur Heart J. 2026 Feb 18;47(34):4826–38. doi: 10.1093/eurheartj/ehag066 (PMC13549725; doi:10.1093/eurheartj/ehag066)
Supplement: Supplementary file 1 [file ehag066_supplementary_data.docx]

**Tropical cyclones and acute coronary syndromes: a Chinese nationwide study**

**Sicheng Li^1^,** **Wei Wang^2^, Hanwen Zhou^3^, Yuqin Zhang^4^, Lin Chen^5^, Jiyi Lin^1,6^,** **Nawsherwan^1^,** **Yong Huo^7^,** **Junbo Ge^8^, Bin Wang^1,6^, Yan Wang^1^**

^1^ Xiamen Cardiovascular Hospital of Xiamen University, School of Medicine, Fujian Branch of National Clinical Research Center for Cardiovascular Diseases, Xiamen, Fujian, China

^2^ Department of Gastroenterology, Xinqiao Hospital, Third Military Medical University, Chongqing, China

^3^ School of Public Health, Chongqing Medical University, Chongqing, China

^4^ Department of Epidemiology & Biostatistics, School of Public Health, Peking University, Beijing, China

^5^ Department of Epidemiology and Health Statistics, West China School of Public Health / West China Fourth Hospital, Sichuan University, Chengdu, Sichuan, China

^6^ Xiamen Chest Pain Quality Control Center, Xiamen, Fujian, China

^7^ Department of Cardiology, Peking University First Hospital, Beijing, China

^8^ Department of Cardiology, Zhongshan Hospital, Fudan University, Shanghai institute of Cardiovascular Diseases, Shanghai, China

**Contents**

**[Supplementary texts](#_Toc213155729)**	[1](#_Toc213155729)

[Text S1 China Chest Pain Center	1](#_Toc213155730)

[Text S2 Variable information	2](#_Toc213155731)

[Text S3 Quality control strategies for Chest Pain Center data	2](#_Toc213155732)

[Text S4 Processing of tropical cyclones (TC) data	4](#_Toc213155733)

[Text S5 Weather station data	5](#_Toc213155734)

[Text S6 Distance from the coastline and GDP data	5](#_Toc213155735)

[Text S7 New-onset or recurrent ACS	6](#_Toc213155736)

**[Supplementary tables](#_Toc213155737)**	[7](#_Toc213155737)

[Table S1 Clinical characteristics of the study population	7](#_Toc213155738)

[Table S2 Influence of excluding individuals on demographic characteristics	9](#_Toc213155739)

[Table S3 102 TCs included in the study	10](#_Toc213155740)

[Table S4 Statistical description of environmental variables	11](#_Toc213155741)

[Table S5 Spearman correlation coefficients of environmental variables	12](#_Toc213155742)

[Table S6 Sensitivity analyses by applying boose model based wind speed	13](#_Toc213155743)

[Table S7 Sensitivity analyses by applying weather station based wind speed	14](#_Toc213155744)

[Table S8 Sensitivity analyses by applying applying hurricane as exposure	15](#_Toc213155745)

[Table S9 Sensitivity analyses by excluding participants with confirmed COVID-19 diagnoses	16](#_Toc213155746)

[Table S10 Sensitivity analyses among individuals with onset and hospital addresses in the same county	17](#_Toc213155747)

[Table S11 Sensitivity analyses by additionally adjusted for PM](#_Toc213155748)_[2.5](#_Toc213155748)_	[18](#_Toc213155748)

[Table S12 Sensitivity analyses by additionally adjusted for O](#_Toc213155749)_[3](#_Toc213155749)_	[19](#_Toc213155749)

[Table S13 Sensitivity analyses by additionally adjusted for the distance from the hospital to the coastline	20](#_Toc213155750)

[Table S14 Sensitivity analyses by removing temperature and humidity	21](#_Toc213155751)

**[Supplementary figures](#_Toc213155752)**	[22](#_Toc213155752)

[Figure S1 Folw chart of the study	22](#_Toc213155753)

[Figure S2 An example of wind field data generation	23](#_Toc213155754)

[Figure S3 The trajectories of 102 TCs included in the study	24](#_Toc213155755)

# Supplementary texts

## Text S1 China Chest Pain Center

The chest pain center is a multidisciplinary, collaborative care system designed for patients with acute chest pain. Through standardized workflows and regional coordination networks, they facilitate rapid recognition and precise treatment of cardiovascular emergencies, particularly acute coronary syndrome, with the primary objective of minimizing delays across the entire “onset-to-reperfusion” timeline. Compared with traditional hospitals, chest pain centers differ in several key respects: 1) Multidisciplinary collaboration. By integrating resources from the emergency department, cardiology, catheterization laboratory, and radiology, chest pain centers form cohesive treatment teams that overcome the siloed operations typical of traditional hospitals and thereby reduce door-to-wire times that are often prolonged by communication gaps and inefficient patient transfers. 2) Standardized processes and time management. The chest pain center implements standardized pathways covering the full continuum from prehospital emergency care to postoperative management (e.g., the “D-to-W pathway” for bypassing the emergency department and proceeding directly to the catheterization laboratory) and closely monitors key time points (e.g., first medical contact-to-ECG acquisition time and troponin draw-to-report time). In contrast, traditional hospitals often have fragmented workflows, leading to treatment delays. 3) Continuous improvement loop. The chest pain center maintains systems for ongoing quality control, optimizing workflows through regular review meetings and dynamic monitoring, while driving long-term improvement via performance incentives and standardized training. Traditional hospitals often lack such structured frameworks and enforcement mechanisms. 4) Comprehensive patient management. The chest pain center extends the treatment chain beyond the hospital to include prehospital emergency care and postdischarge rehabilitation, forming a closed-loop model of “prehospital rapid response—in-hospital precise treatment—out-of-hospital continuous management.” Cooperation agreements with medical alliance units and the 120 emergency centers establish transfer standards and green channel activation mechanisms, creating an integrated urban–rural network that ensures seamless linkage from symptom onset to hospital admission. Standardized follow-up protocols, including scheduled telephone calls, revisit reminders, and rehabilitation counseling, extend patient care from the acute phase to chronic management, reducing the risk of recurrence. In contrast, traditional hospitals are largely confined to isolated in-hospital stages, with marked discontinuities in prehospital emergency and postrehabilitation care, limiting the development of a truly comprehensive treatment advantage.

## Text S2 Variable information

Chinese Cardiovascular Association (CCA) Database–Chest Pain Center provided data on demographic information, risk factors, comorbidities, admission timeline, clinical diagnoses and treatments, and disease progression or outcomes. Demographic information included age, sex, education, and ethnicity. The six risk factors were hypertension, hyperlipidemia, diabetes, smoking, obesity, and a family history of premature CVD. The 13 comorbidities were coronary heart disease, atrial fibrillation, chronic heart failure, valvular heart disease, cerebrovascular disease, peripheral arterial disease, aortic aneurysm, chronic obstructive pulmonary disease, chronic kidney disease, anemia, peptic ulcer, thyroid disorders, and cancer. Comorbidities were ascertained from ICD-10 codes in patients’ discharge diagnoses and include both prior medical history and new diagnoses confirmed by admitting physicians on the basis of examinations and laboratory findings during the current admission.

The admission timeline included the times of symptom onset, arrival at the hospital, physician reception, consultation decision and completion, entry into the catheterization laboratory, and wire crossing, although data on wire crossing time were not available in the present study. Clinical diagnoses and treatments included laboratory indicators, ventricular function, admission diagnosis, initial treatment, revascularization, and discharge diagnosis. Disease progression or outcomes included length of hospital stay, hospitalization costs, and the patient’s final outcome (discharge, transfer to another hospital, transfer to another department, or death).

## Text S3 Quality control strategies for Chest Pain Center data

The completeness and accuracy of data submitted to the CCA Database–Chest Pain Center are supported by several strategies. First, data managers from each register hospital should complete training on definitions of data elements, data collection, data abstraction, and data reporting procedures. All trainees can obtain a working manual of data management. Additional support is available through on-call training and online services from personnel of the headquarter of chest pain centers. In addition, the China Chest Pain Center Congress is held every year by the CCA, China Alliance of Chest Pain Centers, and the headquarter of chest pain centers to provide a platform for networks among registered hospitals, share experiences in data abstraction and reporting, and allow registered hospitals to learn skills to collect high-quality data.

Second, data quality is monitored semi-annually, and review reports are sent to registered hospitals to ensure data completeness. Cases should be enrolled within 7 days once patients receive first medical contact, and online data reporting for cases should be finished within 30 days since the patients are discharged. Hospitals can receive data quality review reports semi-annually and annually. These reports provide detailed information on records that fail to meet enrollment criteria including duplicate case entries, display missing data elements, and possess invalid and illogical values. Registered hospitals need to reconcile these data problems before the CCA Database–Chest Pain Center closes the data record.

Third, the CCA Database–Chest Pain Center sets a series of internal data checks to improve the point-of-entry data quality. Specifically, automatic data check is performed consistently for invalid and illogical values to identify potential errors and trace the relevant records. Most data modules are structured, so that valid data must be entered before the case can be saved as a complete record and reported to the CCA Database–Chest Pain Center.

## Text S4 Processing of tropical cyclones (TC) data

Step 1, download all TC raw data in Network Common Data Form (NetCDF) spanning from 1980 to the present (https://www.ncei.noaa.gov/data/international-best-track-archive-for-climate-stewardship-ibtracs/v04r01/access/netcdf/).

Step 2, process the raw data using the ***defStormsDataset*** function in the ***StormR*** package^1^, which creates a ***stormsDataset*** object from either a NetCDF or CSV file. This step is essential before other StormR functions can be applied. The parameter seasons is set to c(2015, 2022), indicating that TC data from 2015 to 2022 is extracted to create the ***stormsDataset***.

Step 3, employ the ***defStormsList*** function in the ***StormR*** package to filter specific TC data from the ***stormsDataset***. This function extracts storm track data and creates a ***stormsList*** object based on specified arguments, such as the location of interest (***loi***), seasons, and storm names. In this study, ***loi*** is set to China, and ***maxDist*** is set to 300, incorporating all TCs with paths within 300 km of China (illustrated by the blue segments in **Figure S2**) for further analysis. A total of 102 distinct TCs are included.

Step 4, use the ***spatialBehaviour*** function in the ***StormR*** package to analyze individual TC. This function computes wind speed and direction for each cell of a regular grid (i.e., a raster) for a given tropical cyclone or a set of tropical cyclones, and it calculates three associated summary statistics. The parameter ***product*** is set to Profiles, generating 2D wind field data; ***method*** is set to Willoughby, applying the Willoughby wind field model; ***asymmetry*** is set to Chen, utilizing the asymmetry method by Chen et al.; ***tempRes*** is set to 60 minutes; and ***spaceRes*** is set to 10 minutes, representing 18.6×18.6 km at the equator. **Figure S2** depicts TC Ampil’s path and its wind field at 10 p.m. on July 21, 2018.

Step 5, use the ***extract*** function in the ***terra*** package to match the wind field data from Step 4 with hospital addresses, thereby producing wind speed data for each hospital corresponding to a single TC. Repeat Steps 4 and 5 to generate wind speed data for all hospitals and TCs.

Step 6, merge the wind speed data by date and select the maximum value as the daily maximum wind speed to define TC days. Note that multiple TCs may make landfall on the same day, such as TCs SON-TINH and AMPIL simultaneously landing in China on July 21, 2018. However, the primary analysis in this study does not account for the combined wind speed effects of multiple TCs in the same area.

## Text S5 Weather station data

The maximum sustained wind speed data for global meteorological stations were obtained from the National Centers for Environmental Information (<https://w> ww.ncei.noaa.gov/data/global-summary-of-the-day/archive/). Hospitals included in this study were matched to the nearest Chinese meteorological station to collect daily maximum sustained wind speed data from January 1, 2015, to December 31, 2022. To specifically capture TC exposure and minimize the influence of other factors, such as sandstorms, only hospitals affected by TC were included in the analysis.

## Text S6 Distance from the coastline and GDP data

The distance to the coastline is defined as the shortest distance from the hospital to the nearest point on China's coastline. The calculation method is as follows: First, the boundary data of China is obtained using the ***ne_countries*** function from the ***rnaturalearth*** package. Next, the coastline data is extracted using the ***st_cast*** function from the ***sf*** package. The coastline data is then converted into a latitude-longitude matrix using the ***st_coordinates*** function from the ***sf*** package. Finally, the shortest distance is calculated using the ***dist2Line*** function from the ***geosphere*** package. The annual GDP data at the city level were obtained from the China City Statistical Yearbook 2022. The city-level GDP per capita was calculated by dividing the total GDP of each city by its population. These data were then matched to the corresponding hospitals based on their latitude and longitude coordinates.

## Text S7 New-onset or recurrent ACS

We utilized the encrypted ID card number field of ACS cases to distinguish between new-onset and recurrent cases. Records in which this field appeared more than once during the study period (2015–2022) and corresponded to a later admission date were defined as recurrent; all others were classified as new-onset.

In total, this study included 2,563,780 ACS cases, of which 453,443 (18%) lacked an ID card number. Among the 2,110,337 cases (82%) with an ID number, 130,573 admissions were due to recurrence, accounting for only 5.1% of the total sample. Furthermore, because the Chinese Cardiovascular Association began nationwide ACS data collection in 2015 (aligned with our study period), we could not identify patients with a pre-2015 history of ACS who experienced only one recurrence during 2015–2022. We may also have misclassified the first recurrence among patients with pre-2015 ACS who experienced multiple recurrences during the study period as initial onset.

# Supplementary tables

| Table S1 Clinical characteristics of the study population | | | | |
| --- | --- | --- | --- | --- |
| **Characteristic** | **ACS**  **N = 2,563,780** | **STEMI**  **N = 930,378** | **NSTEMI**  **N = 578,083** | **UA**  **N = 1,055,319** |
| ***Laboratory indicators*** |  |  |  |  |
| NT-proBNP, pg/mL | 293 [90-1,317] ***** | 349 [93-1,655] | 770 [197-2,975] | 161 [63-526] |
| CK-MB, ng/mL | 3.8 [2.2-15.1] | 7.2 [2.5-31.6] | 8.4 [3.0-26.4] | 2.5 [1.6-4.0] |
| D-dimer, ng/mL | 1.4 [0.2-227.8] | 2.4 [0.2-240.0] | 3.6 [0.2-280.0] | 0.8 [0.2-200.0] |
| Myoglobin, ng/mL | 43 [25-115] | 68 [30-213] | 71 [30-181] | 30 [20-49] |
| cTNI, ng/mL | 0.10 [0.4-0.75] | 0.28 [0.5-2.90] | 0.46 [0.9-2.45] | 0.5 [0.1-0.10] |
| cTNT, ng/mL | 0.6 [0.4-0.74] | 0.24 [0.4-2.0] | 0.21 [0.5-0.99] | 0.4 [0.1-0.5] |
| Cr, μmol/L | 73.7 [61.2-89.0] | 75.0 [63.0-91.0] | 76.4 [63.4-95.0] | 71.0 [59.3-85.8] |
| ***Ventricular function*** |  |  |  |  |
| LVEF, % | 57.1±10.4 | 54.3±10.3 | 55.8±11.0 | 60.3±9.2 |
| NYHA functional classification |  |  |  |  |
| Class Ⅰ | 1,710,618 (77% **†**) | 653,658 (78%) | 353,572 (74%) | 703,388 (79%) |
| Class Ⅱ | 317,545 (14%) | 108,033 (13%) | 74,160 (16%) | 135,352 (15%) |
| Class Ⅲ | 104,524 (5%) | 32,040 (4%) | 29,343 (6%) | 43,141 (5%) |
| Class Ⅳ | 75,978 (3%) | 45,075 (5%) | 20,765 (4%) | 10,138 (1%) |
| GRACE score | — **‡** | — | 124.1±52.9 | 99.2±48.2 |
| ***Initial Treatment*** |  |  |  |  |
| Anti-platelet therapy | 297,130 (13%) | 42,141 (5%) | 59,636 (13%) | 195,353 (21%) |
| Preoperative anticoagulation | 951,771 (62%) | 293,120 (50%) | 199,037 (60%) | 459,614 (73%) |
| Statin therapy | 501,737 (23%) | 178,583 (22%) | 94,678 (21%) | 228,476 (25%) |
| β-receptor antagonists therapy | 1,094,242 (52%) | 451,240 (59%) | 228,130 (52%) | 414,872 (47%) |
| ***Revascularization*** |  |  |  |  |
| Reperfusion | — | 761,857 (88%) | — | — |
| Reperfusion strategies |  |  |  |  |
| Direct PCI | — | 539,089 (58%) | — | — |
| Thrombolytic therapy | — | 85,567 (9%) | — | — |
| Elective intervention | — | 111,586 (12%) | — | — |
| Others **§** | — | 194,136 (21%) | — | — |
| Treatment strategies |  |  |  |  |
| Conservative Treatment | — | — | 156,620 (33%) | 513,166 (54%) |
| Invasive Therapy | — | — | 315,468 (67%) | 439,851 (46%) |
| Invasive strategies |  |  |  |  |
| Intervention Therapy | — | — | 290,207 (93%) | 273,066 (80%) |
| CABG | — | — | 1,310 (0.4%) | 1,510 (0.4%) |
| Others **\|\|** | — | — | 21,178 (7%) | 65,081 (19%) |
| Initial TIMI flow |  |  |  |  |
| 0 | 280,760 (50%) | 184,852 (69%) | 51,014 (42%) | 44,894 (25%) |
| 1 | 45,839 (8%) | 21,905 (8%) | 12,437 (10%) | 11,497 (7%) |
| 2 | 45,738 (8%) | 18,757 (7%) | 12,469 (10%) | 14,512 (8%) |
| 3 | 194,714 (34%) | 43,836 (16%) | 45,203 (37%) | 105,675 (60%) |
| TIMI flow after revascularization |  |  |  |  |
| 0 | 63,302 (11%) | 17,543 (7%) | 15,569 (13%) | 30,190 (17%) |
| 1 | 3,808 (0.7%) | 1,706 (0.6%) | 1,071 (0.9%) | 1,031 (0.6%) |
| 2 | 10,776 (2%) | 4,945 (2%) | 2,214 (2%) | 3,617 (2%) |
| 3 | 488,459 (86%) | 244,849 (91%) | 102,182 (84%) | 141,428 (80%) |

*******:** Normally distributed continuous variables are presented as the means (standard deviations), skewed distributed continuous variables are presented as medians (interquartile ranges), and discrete variables are presented as n (%). **†:** Because of the existence of missing data, the percentage reflects the proportion of this category relative to all nonmissing values. **‡:** Not applicable to the disease or to its particular subtype. **§:** Undergoing CABG or radiography only. **||:** Undergoing radiography only.

Abbreviations: ACS, acute coronary syndrome; STEMI, ST-segment-elevation myocardial infarction; NSTEMI, non-ST-segment-elevation myocardial infarction; UA, unstable angina; NTproBNP, N-terminal-prohormone brain natriuretic peptide; CK-MB, creatine kinase myocardial band; cTNI, cardiac troponin-I; cTNT, cardiac troponin-T; Cr, creatinine; LVEF: left ventricular ejection fraction; SD, standard deviation; NYHA, New York Heart Association; GRACE, Global Registry of Acute Coronary Events; PCI: percutaneous coronary intervention; CABG, coronary artery bypass graft surgery; TIMI, thrombolysis in myocardial infarction

| Table S2 Influence of excluding individuals on demographic characteristics | | | |
| --- | --- | --- | --- |
| **Demographic characteristic** | **Included**  **N = 3,094,812** | **Excluded**  **N = 531,032** | **Finally-included**  **N = 2,563,780** |
| Age, years | 63.8±12.5 ***** | 62.8±12.9 | 64.0±12.4 |
| Male sex | 2,141,355 (69%) **†** | 397,824 (75%) | 1,743,531 (68%) |
| Education |  |  |  |
| College and above | 42,981 (8%) | 6,048 (7%) | 36,933 (8%) |
| Secondary school | 268,186 (49%) | 44,873 (52%) | 223,313 (48%) |
| Primary school and below | 239,039 (43%) | 36,028 (41%) | 203,011 (44%) |
| Han ethnic group | 1,183,417 (94%) | 181,113 (92%) | 1,002,304 (94%) |

***:** Means (standard deviation).

**†:** N (%).

Demographic characteristics of the included, excluded and finally-included population

| Table S3 102 TCs included in the study | | | | | | | |
| --- | --- | --- | --- | --- | --- | --- | --- |
| 2015 | 2016 | 2017 | 2018 | 2019 | 2020 | 2021 | 2022 |
| Kujira | Unnamed | Mora | Ewiniar | Fani | Amphan | Koguma | Chaba |
| Chan-Hom | Nepartak | Merbok | Gaemi | Mun | Nuri | Unnamed | Songda |
| Linfa | Mirinae | Nanmadol | Maria | Danas | Sinlaku | In-Fa | Unnamed |
| Soudelor | Nida | Talas | Son-Tinh | Wipha | Hagupit | Cempaka | Ma-On |
| Vamco | Dianmu | Sonca | Ampil | Francisco | Jangmi | Lupit | Hinnamnor |
| Dujuan | Meranti | Roke | Unnamed | Lekima | Mekkhala | Omais | Muifa |
| Mujigae | Rai | Nesat | Jongdari | Bailu | Higos | Conson | Noru |
| — | Malakas | Haitang | Yagi | Podul | Bavi | Chanthu | Nesat |
| — | Megi | Hato | Bebinca | Kajiki | Maysak | Lionrock | Sonca |
| — | Chaba | Pakhar | Rumbia | Lingling | Haishen | Kompasu | Unnamed |
| — | Aere | Mawar | Soulik | Mitag | Noul | Rai | Nalgae |
| — | Sarika | Guchol | Unnamed | — | Nangka | — | — |
| — | Haima | Talim | Mangkhut | — | Saudel | — | — |
| — | — | Doksuri | Barijat | — | Atsani | — | — |
| — | — | Unnamed | Kong-Rey | — | Vamco | — | — |
| — | — | Unnamed | Yutu | — | — | — | — |
| — | — | Khanun | — | — | — | — | — |
| — | — | Haikui | — | — | — | — | — |

Abbreviations: TCs, tropical cyclones.

| Table S4 Statistical description of environmental variables | | | | | | | | |
| --- | --- | --- | --- | --- | --- | --- | --- | --- |
| **Variable** | **Mean** | **SD** | **IQR** | ***P*_0.1_** | ***P*_25_** | **Median** | ***P*_75_** | ***P*_99.9_** |
| Wind speed (Willoughby) *****, m/s | 14.3 | 6.4 | 8.8 | 2.7 | 9.3 | 13.2 | 18.2 | 41.4 |
| Wind speed (Boose) *****, m/s | 10.1 | 5.5 | 7.3 | 0.0 | 6.2 | 9.3 | 13.6 | 31.8 |
| Wind speed (station), m/s | 4.5 | 2.2 | 3.1 | 1.0 | 3.0 | 4.0 | 6.0 | 16.0 |
| Temperature, ℃ | 16.0 | 10.8 | 16.2 | -21.3 | 8.5 | 17.7 | 24.8 | 34.7 |
| Humility, % | 68.4 | 18.8 | 26.8 | 11.0 | 56.6 | 72.4 | 83.4 | 98.4 |
| PM_2.5_, μg/m^3^ | 40.1 | 33.4 | 29.1 | 4.4 | 19.5 | 30.6 | 48.6 | 276.0 |
| O_3_, μg/m^3^ | 101.8 | 46.4 | 63.2 | 8.5 | 67.2 | 95.0 | 130.4 | 273.9 |

This results solely reflect scenarios involving ACS admission days.

***:** Wind speed (Willoughby or Boose method) associated with TC, excluding region out of the TC wind field.

Wind speed (Willoughby): Wind speed calculated from Willoughby wind field model; Wind speed (Boose): Wind speed calculated from Boose wind field model; Wind speed (station): Wind speed from the nearest weather station.

Abbreviations: ACS, acute coronary syndrome; SD, standard deviation; IQR, interquartile range; ***P***_0.1_, the 0.1th percentile; ***P***_25_, the 25th percentile; ***P***_75_, the 75th percentile; ***P***_99.9_, the 99.9th percentile; PM_2.5_, fine particulate matter; O_3_, ozone; TC, tropical cyclone.

| Table S5 Spearman correlation coefficients of environmental variables | | | | | | |
| --- | --- | --- | --- | --- | --- | --- |
| Environmental variables | Wind speed  (Willoughby *****) | Wind speed  (Boose *****) | Wind speed (station) | Temperature | Humility | PM_2.5_ |
| Wind speed (Boose) | 0.884 |  |  |  |  |  |
| Wind speed (station) | 0.415 | 0.406 |  |  |  |  |
| Temperature | 0.103 | 0.090 | -0.058 |  |  |  |
| Humility | -0.053 | -0.062 | 0.045 | 0.179 |  |  |
| PM_2.5_ | -0.091 | -0.128 | -0.172 | -0.397 | -0.004 |  |
| O_3_ | -0.105 | -0.104 | -0.116 | 0.588 | -0.154 | -0.170 |

This results solely reflect scenarios involving ACS admission days.

***:** Regions outside the TC wind field were excluded when calculating Spearman correlation coefficients between wind speed (using the Willoughby or Boose methods) and other variables.

Wind speed (Willoughby): Wind speed calculated from Willoughby wind field model; Wind speed (Boose): Wind speed calculated from Boose wind field model; Wind speed (station): Wind speed from the nearest weather station.

All the Spearman correlation coefficients were statistically significant (*P* value < 0.05).

Abbreviations: ACS, acute coronary syndrome; PM_2.5_, fine particulate matter; O_3_, ozone.

| Table S6 Sensitivity analyses by applying boose model based wind speed | | | | | |
| --- | --- | --- | --- | --- | --- |
| Model | Outcome | RR | LCI | HCI | P |
| Main analyses ***** | ACS | 1.135 | 1.018 | 1.265 | 0.022 |
|  | STEMI | 1.308 | 1.126 | 1.521 | <0.001 |
|  | NSTEMI | 1.446 | 1.205 | 1.734 | <0.001 |
|  | UA | 1.136 | 1.023 | 1.261 | 0.017 |
| Sensitive analyses **†** | ACS | 1.181 | 1.025 | 1.359 | 0.021 |
|  | STEMI | 1.177 | 1.012 | 1.368 | 0.034 |
|  | NSTEMI | 1.241 | 1.045 | 1.473 | 0.014 |
|  | UA | 1.126 | 1.012 | 1.254 | 0.030 |

***:** The main analyses represented the analyses in the manuscript. The exposure was TC, defined as Willoughby-model wind speed > 17.5 m/s. The study population comprised 2,563,780 ACS patients. A 14-day lag period was applied. The model included cross-basis terms for TC, temperature, humidity, day of the week, and a natural spline for year. For readability and to enable direct comparison with the sensitivity analysis, we present the main analysis results here.

**†:** Boose model based wind speed data were applied here to assess whether the results are sensitive to the wind field model.

Abbreviations: RR, relative risk; LCI, low confidence interval; HCI, high confidence interval; ACS, acute coronary syndrome; STEMI, ST-segment-elevation myocardial infarction; NSTEMI, non-ST-segment-elevation myocardial infarction; UA, unstable angina; and TC, tropical cyclone.

| Table S7 Sensitivity analyses by applying weather station based wind speed | | | | | |
| --- | --- | --- | --- | --- | --- |
| Model | Outcome | RR | LCI | HCI | P |
| Main analyses ***** | ACS | 1.135 | 1.018 | 1.265 | 0.022 |
|  | STEMI | 1.308 | 1.126 | 1.521 | <0.001 |
|  | NSTEMI | 1.446 | 1.205 | 1.734 | <0.001 |
|  | UA | 1.136 | 1.023 | 1.261 | 0.017 |
| Sensitive analyses **†** | ACS | 1.103 | 1.011 | 1.203 | 0.027 |
|  | STEMI | 1.244 | 1.005 | 1.539 | 0.045 |
|  | NSTEMI | 1.232 | 1.06 | 1.431 | 0.006 |
|  | UA | 1.271 | 1.035 | 1.561 | 0.022 |

***:** The main analyses represented the analyses in the manuscript. The exposure was TC, defined as Willoughby-model wind speed > 17.5 m/s. The study population comprised 2,563,780 ACS patients. A 14-day lag period was applied. The model included cross-basis terms for TC, temperature, humidity, day of the week, and a natural spline for year. For readability and to enable direct comparison with the sensitivity analysis, we present the main analysis results here.

**†:** Weather station based wind speed data were applied to assess the impact of the simultaneous occurrence of multiple TCs on the results.

Abbreviations: RR, relative risk; LCI, low confidence interval; HCI, high confidence interval; ACS, acute coronary syndrome; STEMI, ST-segment-elevation myocardial infarction; NSTEMI, non-ST-segment-elevation myocardial infarction; UA, unstable angina; and TC, tropical cyclone.

| Table S8 Sensitivity analyses by applying applying hurricane as exposure | | | | | |
| --- | --- | --- | --- | --- | --- |
| Model | Outcome | RR | LCI | HCI | P |
| Main analyses ***** | ACS | 1.135 | 1.018 | 1.265 | 0.022 |
|  | STEMI | 1.308 | 1.126 | 1.521 | <0.001 |
|  | NSTEMI | 1.446 | 1.205 | 1.734 | <0.001 |
|  | UA | 1.136 | 1.023 | 1.261 | 0.017 |
| Sensitive analyses **†** | ACS | 1.197 | 1.007 | 1.423 | 0.042 |
|  | STEMI | 1.351 | 1.04 | 1.756 | 0.023 |
|  | NSTEMI | 1.334 | 1.043 | 1.706 | 0.021 |
|  | UA | 1.115 | 1.024 | 1.214 | 0.012 |

***:** The main analyses represented the analyses in the manuscript. The exposure was TC, defined as Willoughby-model wind speed > 17.5 m/s. The study population comprised 2,563,780 ACS patients. A 14-day lag period was applied. The model included cross-basis terms for TC, temperature, humidity, day of the week, and a natural spline for year. For readability and to enable direct comparison with the sensitivity analysis, we present the main analysis results here.

**†:** Hurricane identified by Willoughby model wind speed >33 m/s is applied to evaluate the effect of different wind speed thresholds on the results.

Abbreviations: RR, relative risk; LCI, low confidence interval; HCI, high confidence interval; ACS, acute coronary syndrome; STEMI, ST-segment-elevation myocardial infarction; NSTEMI, non-ST-segment-elevation myocardial infarction; UA, unstable angina; and TC, tropical cyclone.

| Table S9 Sensitivity analyses by excluding participants with confirmed COVID-19 diagnoses | | | | | |
| --- | --- | --- | --- | --- | --- |
| Model | Outcome | RR | LCI | HCI | P |
| Main analyses ***** | ACS | 1.135 | 1.018 | 1.265 | 0.022 |
|  | STEMI | 1.308 | 1.126 | 1.521 | <0.001 |
|  | NSTEMI | 1.446 | 1.205 | 1.734 | <0.001 |
|  | UA | 1.136 | 1.023 | 1.261 | 0.017 |
| Sensitive analyses **†** | ACS | 1.138 | 1.021 | 1.269 | 0.020 |
|  | STEMI | 1.317 | 1.133 | 1.532 | <0.001 |
|  | NSTEMI | 1.451 | 1.209 | 1.741 | <0.001 |
|  | UA | 1.138 | 1.021 | 1.269 | 0.020 |

***:** The main analyses represented the analyses in the manuscript. The exposure was TC, defined as Willoughby-model wind speed > 17.5 m/s. The study population comprised 2,563,780 ACS patients. A 14-day lag period was applied. The model included cross-basis terms for TC, temperature, humidity, day of the week, and a natural spline for year. For readability and to enable direct comparison with the sensitivity analysis, we present the main analysis results here.

**†:** Participants with COVID-19 were excluded to evaluate the effect of incident COVID-19 during the study period on the results, as COVID-19 appears to be associated with ACS^2^.

Abbreviations: RR, relative risk; LCI, low confidence interval; HCI, high confidence interval; ACS, acute coronary syndrome; STEMI, ST-segment-elevation myocardial infarction; NSTEMI, non-ST-segment-elevation myocardial infarction; UA, unstable angina; and TC, tropical cyclone.

| Table S10 Sensitivity analyses among individuals with onset and hospital addresses in the same county | | | | | |
| --- | --- | --- | --- | --- | --- |
| Model | Outcome | RR | LCI | HCI | P |
| Main analyses ***** | ACS | 1.135 | 1.018 | 1.265 | 0.022 |
|  | STEMI | 1.308 | 1.126 | 1.521 | <0.001 |
|  | NSTEMI | 1.446 | 1.205 | 1.734 | <0.001 |
|  | UA | 1.136 | 1.023 | 1.261 | 0.017 |
| Sensitive analyses **†** | ACS | 1.234 | 1.010 | 1.507 | 0.039 |
|  | STEMI | 1.438 | 1.087 | 1.093 | 0.011 |
|  | NSTEMI | 1.606 | 1.146 | 2.253 | 0.006 |
|  | UA | 1.260 | 1.006 | 1.577 | 0.044 |

***:** The main analyses represented the analyses in the manuscript. The exposure was TC, defined as Willoughby-model wind speed > 17.5 m/s. The study population comprised 2,563,780 ACS patients. A 14-day lag period was applied. The model included cross-basis terms for TC, temperature, humidity, day of the week, and a natural spline for year. For readability and to enable direct comparison with the sensitivity analysis, we present the main analysis results here.

**†:** Analyses were restricted to individuals with onset and hospital addresses in the same county to evaluate the effect of potential exposure misclassification on the results.

Abbreviations: RR, relative risk; LCI, low confidence interval; HCI, high confidence interval; ACS, acute coronary syndrome; STEMI, ST-segment-elevation myocardial infarction; NSTEMI, non-ST-segment-elevation myocardial infarction; UA, unstable angina; and TC, tropical cyclone.

| Table S11 Sensitivity analyses by additionally adjusted for PM_2.5_ | | | | | |
| --- | --- | --- | --- | --- | --- |
| Model | Outcome | RR | LCI | HCI | P |
| Main analyses ***** | ACS | 1.135 | 1.018 | 1.265 | 0.022 |
|  | STEMI | 1.308 | 1.126 | 1.521 | <0.001 |
|  | NSTEMI | 1.446 | 1.205 | 1.734 | <0.001 |
|  | UA | 1.136 | 1.023 | 1.261 | 0.017 |
| Sensitive analyses **†** | ACS | 1.142 | 1.025 | 1.274 | 0.017 |
|  | STEMI | 1.322 | 1.137 | 1.536 | <0.001 |
|  | NSTEMI | 1.462 | 1.219 | 1.755 | <0.001 |
|  | UA | 1.124 | 1.015 | 1.245 | 0.024 |

***:** The main analyses represented the analyses in the manuscript. The exposure was TC, defined as Willoughby-model wind speed > 17.5 m/s. The study population comprised 2,563,780 ACS patients. A 14-day lag period was applied. The model included cross-basis terms for TC, temperature, humidity, day of the week, and a natural spline for year. For readability and to enable direct comparison with the sensitivity analysis, we present the main analysis results here.

**†:** PM_2.5_ was included in the model as potential mediator.

Abbreviations: PM_2.5_, fine particulate matter; RR, relative risk; LCI, low confidence interval; HCI, high confidence interval; ACS, acute coronary syndrome; STEMI, ST-segment-elevation myocardial infarction; NSTEMI, non-ST-segment-elevation myocardial infarction; UA, unstable angina; and TC, tropical cyclone.

| Table S12 Sensitivity analyses by additionally adjusted for O_3_ | | | | | |
| --- | --- | --- | --- | --- | --- |
| Model | Outcome | RR | LCI | HCI | P |
| Main analyses ***** | ACS | 1.135 | 1.018 | 1.265 | 0.022 |
|  | STEMI | 1.308 | 1.126 | 1.521 | <0.001 |
|  | NSTEMI | 1.446 | 1.205 | 1.734 | <0.001 |
|  | UA | 1.136 | 1.023 | 1.261 | 0.017 |
| Sensitive analyses **†** | ACS | 1.181 | 1.054 | 1.324 | 0.004 |
|  | STEMI | 1.312 | 1.128 | 1.526 | <0.001 |
|  | NSTEMI | 1.453 | 1.211 | 1.744 | <0.001 |
|  | UA | 1.166 | 1.021 | 1.331 | 0.023 |

***:** The main analyses represented the analyses in the manuscript. The exposure was TC, defined as Willoughby-model wind speed > 17.5 m/s. The study population comprised 2,563,780 ACS patients. A 14-day lag period was applied. The model included cross-basis terms for TC, temperature, humidity, day of the week, and a natural spline for year. For readability and to enable direct comparison with the sensitivity analysis, we present the main analysis results here.

**†:** O_3_ was included in the model as potential mediator.

Abbreviations: O_3_, ozone; RR, relative risk; LCI, low confidence interval; HCI, high confidence interval; ACS, acute coronary syndrome; STEMI, ST-segment-elevation myocardial infarction; NSTEMI, non-ST-segment-elevation myocardial infarction; UA, unstable angina; and TC, tropical cyclone.

| Table S13 Sensitivity analyses by additionally adjusted for the distance from the hospital to the coastline | | | | | |
| --- | --- | --- | --- | --- | --- |
| Model | Outcome | RR | LCI | HCI | P |
| Main analyses ***** | ACS | 1.135 | 1.018 | 1.265 | 0.022 |
|  | STEMI | 1.308 | 1.126 | 1.521 | <0.001 |
|  | NSTEMI | 1.446 | 1.205 | 1.734 | <0.001 |
|  | UA | 1.136 | 1.023 | 1.261 | 0.017 |
| Sensitive analyses **†** | ACS | 1.173 | 1.047 | 1.315 | 0.006 |
|  | STEMI | 1.318 | 1.129 | 1.538 | <0.001 |
|  | NSTEMI | 1.520 | 1.266 | 1.824 | <0.001 |
|  | UA | 1.148 | 1.008 | 1.306 | 0.037 |

***:** The main analyses represented the analyses in the manuscript. The exposure was TC, defined as Willoughby-model wind speed > 17.5 m/s. The study population comprised 2,563,780 ACS patients. A 14-day lag period was applied. The model included cross-basis terms for TC, temperature, humidity, day of the week, and a natural spline for year. For readability and to enable direct comparison with the sensitivity analysis, we present the main analysis results here.

**†:** The distance from the hospital to the coastline was included in the model as potential confounder, as this variable is associated with both TC exposure and the spatial distribution of ACS.

Abbreviations: RR, relative risk; LCI, low confidence interval; HCI, high confidence interval; ACS, acute coronary syndrome; STEMI, ST-segment-elevation myocardial infarction; NSTEMI, non-ST-segment-elevation myocardial infarction; UA, unstable angina; and TC, tropical cyclone.

| Table S14 Sensitivity analyses by removing temperature and humidity | | | | | |
| --- | --- | --- | --- | --- | --- |
| Model | Outcome | RR | LCI | HCI | P |
| Main analyses ***** | ACS | 1.135 | 1.018 | 1.265 | 0.022 |
|  | STEMI | 1.308 | 1.126 | 1.521 | <0.001 |
|  | NSTEMI | 1.446 | 1.205 | 1.734 | <0.001 |
|  | UA | 1.136 | 1.023 | 1.261 | 0.017 |
| Sensitive analyses **†** | ACS | 1.142 | 1.025 | 1.273 | 0.016 |
|  | STEMI | 1.328 | 1.143 | 1.544 | <0.001 |
|  | NSTEMI | 1.447 | 1.207 | 1.736 | <0.001 |
|  | UA | 1.145 | 1.039 | 1.262 | 0.006 |

***:** The main analyses represented the analyses in the manuscript. The exposure was TC, defined as Willoughby-model wind speed > 17.5 m/s. The study population comprised 2,563,780 ACS patients. A 14-day lag period was applied. The model included cross-basis terms for TC, temperature, humidity, day of the week, and a natural spline for year. For readability and to enable direct comparison with the sensitivity analysis, we present the main analysis results here.

**†:** Temperature and humidity were removed from the model to assess the total rather than the direct association between TC and ACS.

Abbreviations: RR, relative risk; LCI, low confidence interval; HCI, high confidence interval; ACS, acute coronary syndrome; STEMI, ST-segment-elevation myocardial infarction; NSTEMI, non-ST-segment-elevation myocardial infarction; UA, unstable angina; and TC, tropical cyclone.

# Supplementary figures

### Figure S1 Folw chart of the study

Abbreviation: ACS, acute coronary syndrome; and CCA, the Chinese Cardiovascular Association.

### Figure S2 An example of wind field data generation

This figure illustrates Typhoon Ampil's trajectory and wind field at 10 p.m. on July 21, 2018. Blue dots represent the typhoon's path, the blue line along China's border marks the buffer zone where only typhoons entering this region are included, and the blue circular area shows the typhoon's wind field at the specified time.

### Figure S3 The trajectories of 102 TCs included in the study

The figure illustrates the trajectories of all TCs that reached mainland China within a 300 km buffer zone between 2015 and 2022. Each black line represents a TC, with a total of 102 TCs depicted in the figure.

Abbreviations: TC, tropical cyclone.

**Reference**

1.	UMR-AMAP. *StormR*. <https://github.com/umr-amap/StormR> (November 27 2024)

2.	Metzler B, Siostrzonek P, Binder RK, Bauer A, Reinstadler SJ. Decline of acute coronary syndrome admissions in Austria since the outbreak of COVID-19: the pandemic response causes cardiac collateral damage. *European Heart Journal* 2020;**41**:1852–1853. doi: <https://doi.org/10.1093/eurheartj/ehaa314>
